# Hypothesis-Based Analysis of Gene-Gene Interactions and Risk of Myocardial Infarction

**DOI:** 10.1371/journal.pone.0041730

**Published:** 2012-08-02

**Authors:** Gavin Lucas, Carla Lluís-Ganella, Isaac Subirana, Muntaser D. Musameh, Juan Ramon Gonzalez, Christopher P. Nelson, Mariano Sentí, the Myocardial Infarction Genetics Consortium, the Wellcome Trust Case Control Consortium, Stephen M. Schwartz, David Siscovick, Christopher J. O’Donnell, Olle Melander, Veikko Salomaa, Shaun Purcell, David Altshuler, Nilesh J. Samani, Sekar Kathiresan, Roberto Elosua

**Affiliations:** 1 Cardiovascular Epidemiology and Genetics, IMIM, Barcelona, Spain; 2 Epidemiology and Public Health Network (CIBERESP), Barcelona, Spain; 3 Department of Cardiovascular Sciences, University of Leicester, Leicester, United Kingdom; 4 Leicester NIHR Biomedical Research Unit in Cardiovascular Disease, Glenfield Hospital, Leicester, United Kingdom; 5 Center for Research in Environmental Epidemiology (CREAL), Barcelona, Spain; 6 IMIM (Hospital del Mar Research Institute), Barcelona, Spain; 7 Pompeu Fabra University, Barcelona, Spain; 8 Cardiovascular Health Research Unit, Departments of Medicine and Epidemiology, University of Washington, Seattle, Washington, United States of America; 9 National, Heart, Lung, and Blood Institute and Framingham Heart Study, Framingham, Massachusetts, United States of America; 10 Cardiology Division, Massachusetts General Hospital, Harvard Medical School, Boston, Massachusetts, United States of America; 11 Department of Clinical Sciences, Hypertension and Cardiovascular Diseases, University Hospital Malmö, Lund University, Malmö, Sweden; 12 Department of Chronic Disease Prevention, National Institute for Health and Welfare, Helsinki, Finland; 13 Psychiatric and Neurodevelopmental Genetics Unit, Massachusetts General Hospital, Boston, Massachusetts, United States of America; 14 Stanley Center for Psychiatric Research, Broad Institute, Cambridge, Massachusetts, United States of America; 15 The Broad Institute of MIT and Harvard, Cambridge, Massachusetts, United States of America; 16 Department of Genetics, Harvard Medical School, Boston, Massachusetts, United States of America; 17 Department of Molecular Biology, Massachusetts General Hospital, Boston, Massachusetts, United States of America; 18 Cardiovascular Research Center and Center for Human Genetic Research, Massachusetts General Hospital and Harvard Medical School, Boston, Massachusetts, United States of America; 19 Department of Medicine, Harvard Medical School, Boston, Massachusetts, United States of America; University of Texas School of Public Health, United States of America

## Abstract

The genetic loci that have been found by genome-wide association studies to modulate risk of coronary heart disease explain only a fraction of its total variance, and gene-gene interactions have been proposed as a potential source of the remaining heritability. Given the potentially large testing burden, we sought to enrich our search space with real interactions by analyzing variants that may be more likely to interact on the basis of two distinct hypotheses: a biological hypothesis, under which MI risk is modulated by interactions between variants that are known to be relevant for its risk factors; and a statistical hypothesis, under which interacting variants individually show weak marginal association with MI. In a discovery sample of 2,967 cases of early-onset myocardial infarction (MI) and 3,075 controls from the MIGen study, we performed pair-wise SNP interaction testing using a logistic regression framework. Despite having reasonable power to detect interaction effects of plausible magnitudes, we observed no statistically significant evidence of interaction under these hypotheses, and no clear consistency between the top results in our discovery sample and those in a large validation sample of 1,766 cases of coronary heart disease and 2,938 controls from the Wellcome Trust Case-Control Consortium. Our results do not support the existence of strong interaction effects as a common risk factor for MI. Within the scope of the hypotheses we have explored, this study places a modest upper limit on the magnitude that epistatic risk effects are likely to have at the population level (odds ratio for MI risk 1.3–2.0, depending on allele frequency and interaction model).

## Introduction

Coronary heart disease (CHD) is a leading cause of death and disability worldwide [1], and is characterized by significant heritability [2]. While genome wide association (GWA) studies have identified several genetic markers associated with CHD risk and cardiovascular risk factors, the observed effect sizes of these variants are generally smaller than may have been expected, and account for only a small fraction of the variance in disease risk (e.g. ∼10% for CHD [3]). The marked familial clustering we observe in the general population suggests the presence of heritable risk factors with large effects that GWAS are not designed to detect, such as rare variants or gene-gene interactions (epistasis), among others [4].

The GWAS approach has had some success for most complex phenotypes studied to date thanks to the fact that at least some loci have sufficiently strong risk effects to overcome the multiple-testing burden. However, it seems likely that true epistasis effects will constitute a much smaller fraction of the potential search space than for single loci, and this problem is exacerbated by the fact that the compound genotypes that carry additional risk through interaction will generally be less frequent, since their frequency is the product of that of their component single-locus genotypes (under linkage equilibrium). Our success in detecting these interaction effects relies on their being strong enough to overcome the decrease in power due to their lower frequencies and the amplified multiple testing burden. As for single-locus variants before the beginning of the GWAS era, the likely range of risk effects that gene-gene interactions might have is unknown *a priori* for complex phenotypes.

Given the practical challenges of carrying out epistasis analyses on GWAS data, various statistical approaches to this problem have been proposed and computational tools developed (reviewed by Cordell [5]). Of the relatively limited number of attempts that have been made to address this question at a genome-wide level for complex phenotypes, the majority have tried to alleviate the multiple-testing burden by searching for interactions among a reduced number of variants that are considered more likely to interact on the basis of some hypothesis, such as pathway-directed or candidate variant approaches (e.g. [6], [7], [8]), or have otherwise attempted to minimize the computational burden (e.g. [9], [10]). As far as we are aware, these efforts have not resulted in the discovery of robust gene-gene interaction effects for any of the complex phenotypes investigated.

In this study, we addressed the hypothesis that gene-gene interactions contribute to the risk of early-onset myocardial infarction (MI). From a genome-wide panel, we selected single nucleotide polymorphisms (SNPs) that were hypothesized to be more likely to modulate risk of MI through interaction because (i) they have been shown to be robustly associated with cardiovascular risk factors (CVRF) or clinical endpoints (CHD or MI), or because (ii) they show weak marginal association with MI. We tested for pair-wise interaction between these SNPs to attempt to identify epistatic effects that could partly explain the missing heritability of MI risk.

## Methods

**Figure 1:**
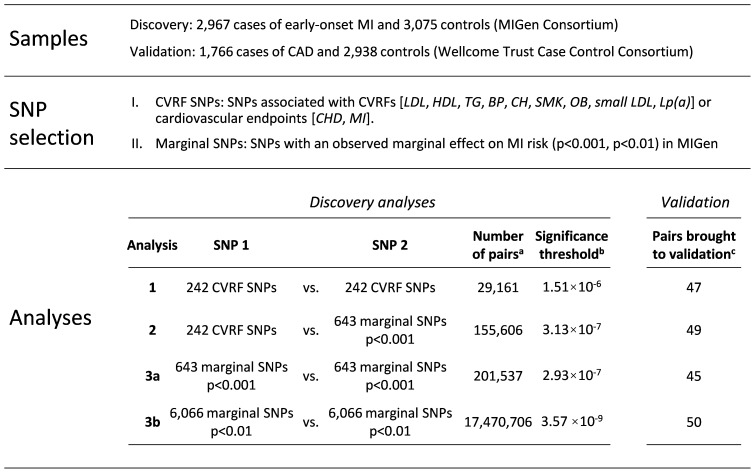
Summary of subjects, methods and analyses. a. Number of SNP pairs for which interaction testing was performed - may not equal the number of possible pair-wise tests [n*(n−1)/2] because some pairs were captured in previous Analyses (File S1 Supporting Figure 2), and some tests were not feasible due to low allele frequencies (File S1 Section 3.3). b. Significance threshold computed using permutations under the null hypothesis (see File S1 Section 3.4) c. SNP pairs with p-value for interaction within 3 orders of magnitude of the significance threshold for each Analysis were brought forward for validation in the WTCCC sample; the numbers of SNP pairs for which data were available in the WTCCC study are shown. *LDL*, concentration of LDL cholesterol; *HDL*, concentration of HDL cholesterol; *TG*, triglyceride concentration; *BP*, blood pressure; *CH*, carbohydrate metabolism (loci associated with risk of Type II diabetes and related phenotypes, such as fasting glucose concentration); *SMK*, smoking; *OB*, obesity; *small LDL*, concentration of small atherogenic LDL particles; *Lp(a)*, plasma levels of lipoprotein(a); *CHD*, risk of coronary heart disease; *MI*, risk of myocardial infarction.

A general outline of the design of this study is shown in Figure 1. An extended description of the methods is given in File S1, and summarized below. Sections, tables and figures in File S1 are indicated in parentheses throughout the manuscript (File S1 Section X.X, Supporting Table X and Supporting Figure X, respectively).

**Figure 2:**
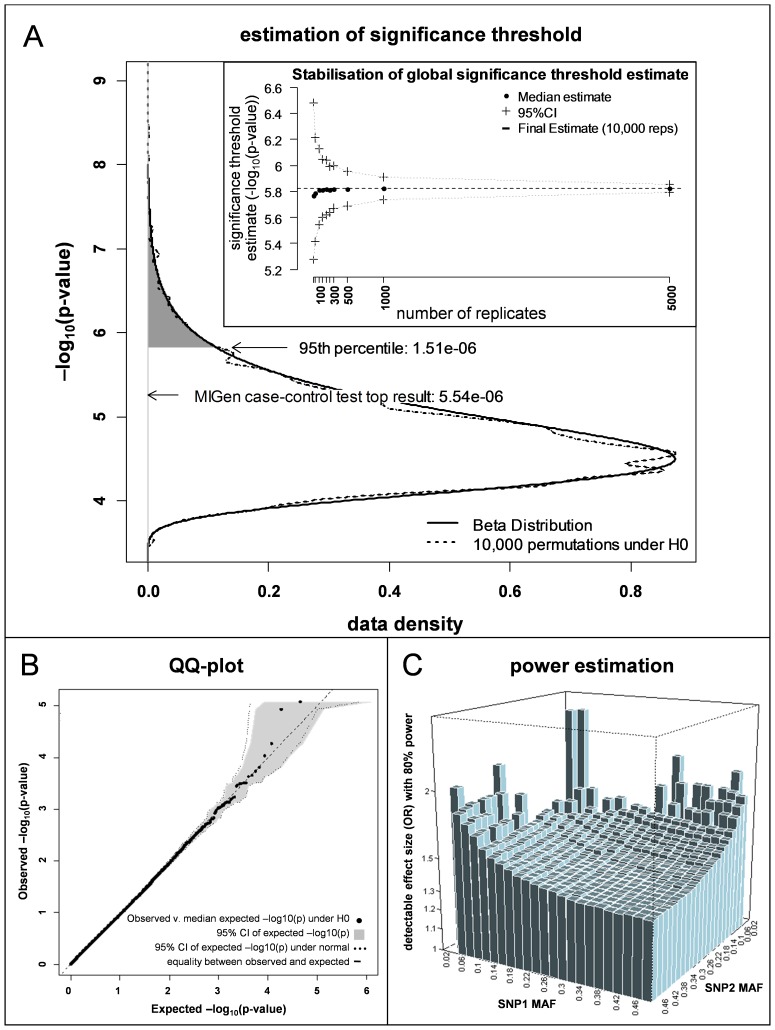
Results of gene-gene interaction search among CVRF SNPs (Analysis 1). *Panel A*. Plot of the top result (arrow) from Analysis 1 against the distribution of the top results from 10,000 permutations under the null hypothesis (dotted line). The permuted top results are expected to follow a beta-distribution (solid line, parameters obtained from permuted top results), the 95^th^ percentile of which was taken as the significance level required to obtain a Type II error of 0.05 (arrow). *Inset*: While the significance level computed in Analysis 1 (dashed black line) was estimated using 10,000 null permutations, this estimate was found to stabilize rapidly with increasing number of permutations (black points) and to change little after 100–200 permutations. Consequently, we progressively reduced the number of permutations used to estimate the significance level in subsequent Analyses. *Panel B*. Quantile-quantile plot showing rank-ordered observed results (black points) from 29,161 tests in Analysis 1 (*y-axis*) against expected results (*x-axis*) estimated from 10,000 permutations under the null hypothesis (randomized phenotype). See File S1 Section 3.5 for computation methods. The shaded area corresponds to the 95%CI of the permuted expected results. The 95%CI of a normal distribution is indicated by the dotted lines. *Panel C*. Estimation of the interaction effect sizes this analysis has 80% power to detect across a range of MAF under an additive × additive interaction model. The heights of the vertical bars correspond to the effect size (OR) detectable for a typical pair of SNPs whose MAFs are as indicated on the horizontal axes.

### Ethics Statement

This study was approved by the Clinical Research Ethics Committee of Parc de Salut MAR, Barcelona.

### Study Design and Subjects (File S1 Section 1)

In this study we performed a discovery analysis of gene-gene interactions using genotype data from the Myocardial Infarction Genetics Consortium (MIGen) [11], consisting of 2,967 cases of early-onset myocardial infarction (men ≤50 or women ≤60 years old), diagnosed on the basis of autopsy evidence, a combination of chest pain and electrocardiographic evidence, or elevation of cardiac biomarkers, and 3,075 age- and sex-matched controls. We validated our top results in a sample of participants from the Wellcome Trust Case Control Consortium [12], consisting of 1,766 cases with a validated history of either MI or coronary revascularization (coronary artery bypass surgery or percutaneous coronary angioplasty) before the age of 66, and 2,938 controls. Genome-wide genotype data and associated phenotype data for the MIGen sample was obtained via The Database of Genotypes and Phenotypes (dbGaP; http://dbgap.ncbi.nlm.nih.gov; project number #2120). All participants gave written informed consent to be included in these studies [11], [12].

### SNP Selection (File S1 Section 2)

We selected SNPs for interaction testing under two hypotheses: i) interactions that modulate MI risk are more likely to occur between SNPs that are individually associated with CVRFs *or* cardiovascular clinical endpoints (hereafter called CVRF SNPs) than between SNPs that are not known to be associated with these phenotypes; ii) SNPs that modulate MI risk via interaction with other SNPs will show at least a low level of marginal association with MI in the MIGen study (hereafter called marginal SNPs).

#### CVRF SNPs (File S1 Sections 2.1–2.3)

We identified SNPs reported to be associated with CVRFs or cardiovascular clinical endpoints by filtering the NHGRI catalogue of GWA studies [13] and mining data from a series of recently published large meta-analyses of GWA studies (see File S1 Supporting Figure 1 for a summary of the literature search and SNP selection process, and File S1 Supporting Table 1 for references). From these studies we identified SNPs that were strongly associated (p<5×10^−8^) with the reported phenotype, and grouped these into 10 categories broadly definable as distinct CVRFs or cardiovascular endpoints (File S1 Supporting Figure 1). These were LDL cholesterol, HDL cholesterol, Triglycerides, Smoking, Blood Pressure, Carbohydrate Metabolism (including Type 2 Diabetes and related traits, e.g. fasting glucose), Obesity/Body Mass, Plasma LP(a) levels, LDL particle size (SNPs reported to be associated with relative concentrations of small, atherogenic LDL particles [14]), and Myocardial Infarction or Coronary Heart Disease (File S1 Supporting Table 1). MI/CHD-associated variation in the *LPA* gene was represented by the haplotypes reported by Trégouët *et al*. [15] (File S1 Section 3.7, File S1 Section 9).

#### Marginal SNPs (File S1 Section 2.4)

We also selected SNPs that achieved an arbitrary p-value of ≤10^−3^ or ≤10^−2^ for association with MI in the MIGen study.

We tested for interaction among CVRF SNPs (Analysis 1), between CVRF SNPs and SNPs with moderate marginal association with MI (p≤10^−3^; Analysis 2), and among SNPs with moderate to weak marginal association with MI (p≤10^−3^, Analysis 3a; p≤10^−2^, Analysis 3b) (Figure 1; File S1 Supporting Figure 2).

### Statistical Analysis (File S1 Section 3)

Interaction analyses were performed using two different tests (File S1 Section 3.3). In Analyses 1–2, we used a case-control test that assumed no specific interaction model but simply contrasted the frequencies of the 9 two-locus genotypes in cases to those in controls [16] by comparing the fits of logistic regression models with and without interaction terms (Test A). We also used this test to verify the results for the most significant interactions from all Analyses in a validation sample from the WTCCC, and performed a fixed effects meta-analysis of both studies (File S1 Section 3.8). To test for interactions between Lp(a) haplotypes and other SNPs, we implemented the same testing framework in a model that also accounts for ambiguous haplotype assignment (File S1 Section 3.7). Due to the greater computational burden of Analyses 3a and 3b, we used a more approximate but faster allelic interaction test implemented in PLINK [17], which compares the correlation between alleles among cases to that among controls (Test B); as a result of the genotype collapsing procedure used in this test and the low minor allele frequencies (MAF) of some SNPs, some SNP pairs could not be compared using this test (File S1 Section 3.3). We also performed case-control/case-only and logic regression analyses as alternative approaches to searching for epistasis in these data; the methods and results of these analyses are detailed in File S1 Notes 1 and 2, respectively.

Within each Analysis, we tested for interaction only between SNPs that were mutually independent (LD r^2^<0.5; File S1 Section 3.2); we also avoided redundancy between Analyses by eliminating SNPs that were in LD (r^2^≥0.5) with SNPs from a previous Analysis (File S1 Section 3.2). We accounted for multiple testing within each Analysis by setting the threshold required to declare a significant result as the 95^th^ percentile of the expected distribution of the most significant p-value for all interaction pairs [18] under the null hypothesis that two-locus compound genotypes do not modify MI risk. These top results follow a beta distribution, the parameters of which were estimated by performing up to 10,000 permutations of each analysis with randomized MI status (Figure 2; File S1 Section 3.4, File S1 Supporting Figure 3). For the purposes of creating quantile-quantile plots, we used the permuted results to compute the expected distribution of ranked test results under the null hypothesis (Figure 2; File S1 Section 3.5, File S1 Supporting Figure 3). We expressed the power of our study in terms of the interaction effect sizes it has 80% power to detect. This was calculated for SNP pairs representing the entire range of observed MAFs and for various interaction models (Figure 2; File S1 Section 3.6, File S1 Supporting Figure 3; and Discussion); the results of the power calculations under an additive × additive interaction model are indicated below for each Analysis. All statistical analyses were carried out using packaged or custom functions written in R v2.11 (R Foundation for Statistical Computing, Vienna [19]), or using PLINK v1.07 [17], where indicated. This report adheres to the recommendations of the STREGA statement on the reporting of genetic association studies [20]; the GWAS studies on which this work is based were completed and/or published before this statement was released, and represent rigorous work reported in a manner consistent with these recommendations.

## Results

### Analysis 1, Tests for Interaction among CVRF SNPs

From the literature sources described above, we identified 242 independent SNPs reported to be robustly associated with CVRFs or cardiovascular endpoints; these SNPs, the reported phenotypes, and the p-values for association with MI in the MIGen study are shown in File S1 Supporting Table 1. Using Test A, we performed 29,161 pair-wise interaction tests among these 242 risk factor SNPs (File S1 Supporting Figure 2), the results of which did not deviate significantly from their empirical expected distribution (Figure 2b). The most significant interaction (p = 5.54×10^−6^; see File S1 Supporting Table 1) occurred between SNPs originally reported to be associated with LDL cholesterol levels (rs2072183, in *NPC1L1*) and smoking initiation (rs1013442, near *BDNF*). This result did not exceed the significance threshold for this Analysis (p = 1.51×10^−6^; Figure 2a; File S1 Supporting Table 2). Under an interaction model with additive × additive effects, we estimated that this analysis had high power (80%) to detect an odds ratio (OR) for interaction of between ∼1.6 and ∼1.3 when both SNPs have a MAF of ∼0.2 and ∼0.5, respectively (Figure 2c; File S1 Supporting Table 3, File S1 Supporting Figure 4).

### Analysis 2, Tests for Interaction between CVRF SNPs and Marginal SNPs (p≤10^−3^)

We selected 656 independent SNPs that showed moderate marginal association (p≤10^−3^) with MI in the MIGen study and excluded 13 that had been captured in Analysis 1. Using Test A, we performed 155,606 interaction tests between the remaining 643 SNPs and the 242 CVRF SNPs (File S1 Supporting Figure 2), the results of which did not deviate significantly from their empirical expected distribution (File S1 Supporting Figure 3). The most significant result for interaction was p = 9.48×10^−7^, between SNPs associated with HDL cholesterol levels (rs3136441, in *LRP4*) and MI (rs9990208, located near *RFTN1* and *DAZL* on chromosome 3, p = 1.2×10^−4^ in MIGen). This result did not exceed the significance threshold for this Analysis (p = 3.13×10^−7^; File S1 Supporting Table 2, File S1 Supporting Figure 3). Under an additive × additive interaction model, this analysis was estimated to have high power to detect interaction effects of between ∼1.7 and ∼1.4 for SNPs with MAF of ∼0.2 and ∼0.5, respectively (File S1 Supporting Table 3, File S1 Supporting Figure 4).

### Analysis 3a, Tests for Interaction among Marginal SNPs (p≤10^−3^)

For the 643 independent SNPs that achieved a p-value of ≤10^−3^ for association with MI in the MIGen study and that were not captured in Analysis 1, we performed 201,537 pair-wise interaction tests using Test B (out of a possible 206,403 pairs; test not feasible for 4,866 pairs (∼2.35%) due to low allele frequencies, see File S1 Section 3.3, File S1 Supporting Figure 2). The results of these tests did not deviate significantly from their empirical expected distribution (File S1 Supporting Figure 3). The most significant p-value for interaction was 3.49×10^−6^, between rs761174 (within *HHAT* on chromosome 1, p = 1.75×10^−5^ in MIGen) and rs167490 (within *CHST11* on chromosome 12, p = 5.92×10^−4^ in MIGen), which did not exceed the significance threshold for this Analysis (p = 2.93×10^−7^; File S1 Supporting Figure 3c). Under an additive × additive interaction model, this analysis was estimated to have high power to detect interaction effects of between ∼1.75 and ∼1.4 for SNPs with MAF of ∼0.2 and ∼0.5, respectively (File S1 Supporting Table 3, File S1 Supporting Figure 4).

### Analysis 3b, Tests for Interaction among Marginal SNPs (p≤10^−2^)

Relaxing the minimum threshold of the observed marginal effects of putative interacting SNPs, we selected 6,066 independent SNPs that achieved a p-value of ≤10^−2^ for association with MI in the MIGen study and that were not captured in the previous Analyses, and performed 17,470,706 interaction tests, out of a possible 18,180,305 pairs (discarded 214,840 tests already captured by previous Analyses; test not feasible for a further 709,599 (∼3.9%) pairs due to low allele frequencies, see File S1 Section 3.3, File S1 Supporting Figure 2). The results of these tests did not deviate significantly from their empirical expected distribution (File S1 Supporting Figure 3). The most significant p-value for interaction was 5.51×10^−8^, between rs194243 (between *CYP26B1* and *EXOC6B* on chromosome 2, p = 3.97×10^−3^ in MIGen) and rs4589969 (within *CACNA2D3* on chromosome 3, p = 7.75×10^−3^ in MIGen), which did not exceed the significance threshold for this Analysis (p = 3.57×10^−9^; File S1 Supporting Figure 3d). Under a double-additive model, this analysis was estimated to have high power to detect interaction effects of between ∼1.85 and ∼1.45 for SNPs with MAF of ∼0.2 and ∼0.5, respectively (File S1 Supporting Table 3, File S1 Supporting Figure 4).

### Validation of the Top Results from Analyses 1–3 in an Independent Sample

While the minimum observed p-values in each Analysis were ∼3–15 times larger than the corresponding significance threshold, it is possible that real interaction effects are present but could not be declared statistically significant because of the demanding multiple testing burden. Therefore, we sought to validate our findings for all SNP pairs that achieved a p-value for interaction within 3 orders of magnitude of the required significance threshold in each Analysis (File S1 Section 3.8). In a large sample of cases of CHD and controls from the WTCCC (File S1 Section 1), we replicated our analysis for 47, 49, 45 and 50 pairs of SNPs (out of 48, 52, 54 and 55 pairs that met this criterion) in Analyses 1, 2, 3a and 3b, respectively. After correcting for multiple testing, none of these pairs showed nominally significant evidence of interaction in the WTCCC data (File S1 Supporting Table 2) for the SNP pairs from Analysis 1 (p_min_ = 0.0041; α≈0.05/47≈0.0011), Analysis 2 (p_min_ = 0.0392; α≈0.05/49≈0.001), Analysis 3a (p_min_ = 0.006; α≈0.05/45≈0.001) or Analysis 3b (p_min_ = 0.012; α≈0.05/50≈0.001). Similarly, we observed no additional evidence of interaction after performing a meta-analysis of both studies (see File S1 Section 3.8 for methods and File S1 Supporting Table 2 for results; Analysis 1, p_min_ = 1.49×10^−5^; Analysis 2, p_min_ = 1.41×10^−5^; Analysis 3a, p_min_ = 1.01×10^−4^; Analysis 3b, p_min_ = 7.01×10^−7^; significance thresholds equal to those for the corresponding discovery Analyses, p = 1.51×10^−6^, p = 3.13×10^−7^, p = 2.93×10^−7^, p = 3.57×10^−9^, respectively).

## Discussion

In this paper, we searched for gene-gene interactions that modify MI risk. Given the potentially large testing burden [21], we sought to enrich our search space with real interactions by analyzing variants that may be more likely to interact on the basis of two distinct hypotheses: i) a biological hypothesis, under which MI risk is modulated by the relationships between variants that are known to be relevant for its risk factors or for MI/CHD risk directly; and ii) a statistical hypothesis, under which the marginal effects of true interactions are detectable as weak single locus associations. We performed pair-wise SNP interaction testing in three Analyses, first requiring that both potentially interacting SNPs have highly significant effects on CHD risk or CV risk factors (Analysis 1), then relaxing this requirement for one of the SNPs, requiring that it be at least moderately associated with MI (p≤0.001; Analysis 2), and finally requiring that both SNPs have only moderate (p≤0.001) or weak (p≤0.01) marginal association with MI (Analyses 3a and 3b, respectively). No evidence for interaction beyond that allowed for by chance was observed in any of these analyses, and we observed no clear consistency between the results for the most significant interactions in the discovery sample and those in a large validation sample. Our study is among the first to use GWAS data to investigate the role of epistasis as a potential risk factor for myocardial infarction and related phenotypes [22], [23].

The search for epistatic effects that are relevant for complex phenotypes can be, and has been, approached using a range of strategies, more so than has been necessary for single-locus GWA studies. Our study is not an exhaustive genome-wide search, but is limited to two hypotheses about the nature of epistasis as a risk factor for MI.

In relation to the first hypothesis, it seems reasonable to assume that interactions that modulate cardiovascular risk are more likely to occur between genes that are known to be relevant for cardiovascular function than those that are not. A similar reasoning formed the basis of a recent study of the role of epistasis in modifying HDL cholesterol levels, in which the authors searched for interactions between genes that were not only known to be relevant for cardiovascular function but that also lie within the same metabolic pathways [6]. Similarly, the most significant gene-gene interactions reported in a recent study of diabetes occurred between variants that were previously shown to be directly associated with diabetes risk [24]. Nonetheless, we note a potential bias against finding interactions under this hypothesis due to the fact that the initial discovery and replication of direct associations between these SNPs and CVRFs might have been possible precisely because these associations are not modulated by interactions. However, given that the response phenotype in this study is MI/CHD, the effect of this bias is probably limited to the CHD-associated SNPs. A further limitation of our analysis under the first hypothesis is the fact that we selected only the most robustly associated CVRFs SNPs, so our analysis does not explore potential interactions between other variants at these loci.

In relation to the second hypothesis, even in the absence of a main effect, a SNP that modifies disease risk through interaction must necessarily show some level of marginal risk effect because some individuals will also carry the interacting allele of the other SNP. This is true for all interaction models except those between SNPs with balanced opposing effects on risk [25]; we believe that such models are unlikely to be an important contributor to heritability of complex phenotypes (but see ref [26]), and our inability to detect them is compensated for by the increase in power due to the reduced multiple testing burden. However, while the reasoning for selecting SNPs with marginal effects is valid, it is not clear how strong these marginal effects might be. Moreover, they are expected to vary as a function of the mode of interaction (e.g. dominant × dominant), and the frequencies and effect sizes of the risk-associated compound genotypes. The thresholds of marginal effect used in this study have been arbitrarily selected for practical purposes.

In this study, interaction analyses were performed by using a logistic regression framework to search for differences in the distributions of the nine two-locus compound genotypes between cases and controls, thereby assuming no specific interaction model. Since the putative true interaction model is unknown, this model-free approach aims to maximize power by encompassing all possible interaction models, while requiring only one test for each SNP pair; this gain in scope is expected to offset the loss of power due to the additional degree of freedom. At any rate, for the top SNP pairs from all three Analyses we also tested for interaction under biologically meaningful interaction models (dominant × dominant, recessive × recessive and additive × additive, data not shown), which, if true, would have higher power under H1, and found no remarkably different results compared to those in which no interaction model was assumed. In Analyses 1 and 2 we also implemented a case-control/case-only test, which has previously been reported to have higher power than the design we have chosen; however, we observed very similar results for both designs (see File S1 Note 1).

The negative results observed in our study may reflect one of two possibilities. First, the role of gene-gene interactions in CHD risk is negligible; this is contrary to the expectations of some authors [27], and does seem unlikely given the complexity of biological systems and the opportunities for interaction within and between genes and pathways that are relevant for disease risk. Conversely, the observation that interactions do not greatly modify CHD risk would be consistent with the extensive redundancy observed in biological systems, and with a recent study that indicated the potential role of random variation in gene expression in modulating the penetrance of interaction effects [28]. This possibility is also consistent with the results of a study that provided theoretical and experimental data to suggest that most of the variance in complex traits is accounted for by additive effects [29], and also possibly by the fact that genetic load, often represented as a genetic score in the context of the results of GWAS studies, has been observed to have generally linear effects on a range of diseases and traits [30], [31], [32], [33].

The second possible explanation for the negative results in this study concerns the limitations of its design or size. The cross-sectional nature of the MIGen study carries an inherent possibility of survival bias, and although the early age of onset is expected to enrich the cases' genetic risk, this effect might be diluted by the fact that the controls are age-matched and may represent future cases. In addition, the WTCCC controls are represented by blood donors whose CHD status may not have been well known, and by recruits from the relatively young 1958 birth cohort who may yet develop CHD. Moreover, given the large sample sizes required to detect recently discovered single loci associated with MI/CHD [3], our study might be expected to have limited power to detect even more complex and potentially more subtle epistasis effects. However, we estimate that this study is well powered to detect interaction effects of a magnitude only slightly larger than those observed for single loci in GWA studies (OR between 1.3 and 2, depending on the interaction model and MAFs; see File S1 Supporting Table 3, File S1 Supporting Figure 4 and below). At a minimum, our results do not support the existence of strong epistasis effects as a common risk factor for CHD. The clinical utility of data on interactions with more subtle effects may be limited and would be difficult to detect with current strategies. Nonetheless, this does not preclude the possibility that a very large number of weaker and/or rarer interactions could account for a significant portion of the variance in disease risk.

In this study, we used two approaches to distinguish between true and false positives, as recommended by Yang *et al*. [18]. First, we compared the p-values observed in our discovery sample to their expected null distribution, which is a non-standard distribution due to the non-independence of the tests. The top result of a large number of tests is known to follow a beta distribution, whose parameters can be estimated by performing a large number of permutations of all tests under the null hypothesis (randomized case status), and taking the top result from each. The significance level corresponding to the desired false positive rate within each Analysis, taken as 0.05 in this study, is then equal to the 95^th^ percentile of this beta distribution. We found that these parameters stabilized after 200–300 permutations, which was practical when using the –fast-epistasis function in PLINK [17], even for the computationally intensive 3rd stage of analysis (∼1.7×10^7^ tests). This empirical approach to determining the global significance level should be feasible for epistasis studies with even broader scope than ours, such as that reported by Bell *et al*. [24], who searched for interactions among ∼70,000 tag-SNPs as a potential risk factor for Type 2 diabetes. We note, however, that in all Analyses the computed empirical significance levels were very similar to those obtained using a Bonferroni correction (File S1 Section 7), suggesting that, at least in this study, little power would be lost by assuming independence between tests. In fact, since any one SNP will be uncorrelated with the vast majority of other SNPs, and since we have specifically taken steps to ensure linkage equilibrium between SNPs in each Analysis, it seems possible that an initial assumption of independence between tests would be entirely reasonable.

Second, we sought to validate the top results from our discovery analysis in an independent sample. This approach essentially represents a biological validation of the results in the discovery stage, and is potentially useful when real effects are present but are too subtle to overcome the multiple testing burden; consistent, but not necessarily globally significant, results in the discovery and validation analyses would constitute qualitative evidence for epistasis that could be pursued more directly. This approach was recently used as an alternative to the permutation-based approach in order to verify the top results of a discovery analysis of gene-gene interactions in relation to HDL cholesterol levels [6]. However, having observed entirely negative results in our discovery analysis, deciding which strategy to use to select interaction pairs to bring forward for validation is largely arbitrary.

To facilitate interpretation of our results, we performed post-hoc power calculations. The most important determinants of the power of any epistasis analysis are the true interaction effect sizes, the interaction model, the frequency of the interacting variants and the strength of LD between the true and observed variants, all of which (apart from the latter, see below) are naturally determined and beyond the control of experimental design. While overall power can be optimized through modifiable determinants of power, such as sample size and analytical strategy, any analysis will have greater power to detect some epistasis models than others. Given that the likely range of true interaction effect sizes is unknown, we expressed the power of our analysis as the minimum interaction effect size we had high power to detect (Type II error  = 0.2), and performed this computation under various interaction models and across a grid of allele frequency combinations (Figure 2c, File S1 Supporting Table 3, File S1 Supporting Figure 4). While many different interaction models are possible [25], our aim was to assess the range of power our study might have under the intrinsically least powerful and most powerful models, recessive × recessive, and additive × additive, respectively (we also computed power under a dominant × dominant model). For each interaction model, and throughout the range of MAFs, these computations result in a characteristic 'surface' of effect sizes (Figure 2c, File S1 Supporting Figure 4), at and above which our study has high power to detect interactions. Moreover, note that although power is lower for effect sizes below this surface, weaker interactions could still be detected; for example, our study has 50% power to detect effect sizes ∼13% lower than those we have 80% power to detect, and we might expect to discover approximately half of such interactions. Finally, one of the most important determinants of power that falls within the control of experimental design to some extent is the strength of LD between the true causal interacting variants and those analyzed. This is important for epistasis analyses because increasing LD attenuates power to detect non-additive effects faster than for additive effects (e.g. [16]), such that complex effects may be essentially undetectable unless they are captured with near-perfect LD. In this sense, the LD pruning steps we have taken to minimize redundant testing may actually have a greater negative effect on power than the gain in power afforded by reducing the multiple-testing burden. For the same reason the (non-additive) interaction model-free testing framework (Test A) may have lower power than the additive test (Test B), in addition to the power reduction due to the additional degrees of freedom.

While this study was limited to testing second-order interactions, as a preliminary exploration we performed a logic regression analysis [34], which searches for higher order interactions using an adaptive regression methodology, and which also provides information on the level of interaction complexity that might best explain the observed data (File S1 Note 2). The results of this analysis generally indicated that little or no additional gain of information was expected by exploring pair-wise, 3-, 4- or 5-way interactions. These results are consistent with those of our main analyses, indicating that higher order interactions may not play an important role in population-wide disease risk population level. At any rate, an exhaustive search for higher-order interactions was, of course, beyond the scope of this study, as it would be for most 'genome-wide' epistasis studies.

### Conclusions

We have performed an extensive search for interactions between SNPs that are robustly associated with classical CVRFs, or that show marginal association with MI. Our results and post-hoc power computations do not support the existence of strong interaction effects as a common risk factor for MI in the general population. This is consistent with the expectation that epistasis effects will be difficult to detect for complex diseases. Within the scope of the hypotheses we have explored, this study places a modest upper limit on the risk effect sizes gene-gene interactions are likely to have (OR for MI risk ∼1.3–2.0, depending on allele frequency and interaction model). While the discovery of gene-gene interactions could provide important insights into molecular function, considering the generally lower frequency and greater complexity of these modest effects, their usefulness in driving drug discovery and especially in improving cardiovascular risk assessment may be limited.

## Supporting Information

File S1 **Supporting Information.** An extended description of the methods and results, plus supplementary tables, figures, notes, appendices and references.
(PDF)
