# Supplementary material for: Hypothesis-Based Analysis of Gene-Gene Interactions and Risk of Myocardial Infarction
Source: PLoS One. 2012 Aug 2;7(8):e41730. doi: 10.1371/journal.pone.0041730 (PMC3410908; doi:10.1371/journal.pone.0041730)
Supplement: File S1 — Supporting Information. An extended description of the methods and results, plus supplementary tables, figures, notes, appendices and references. (PDF) [file pone.0041730.s001.pdf]

SUPPORTING INFORMATION

Hypothesis-based analysis of gene-gene interactions in risk of myocardial infarction

Gavin Lucas MSc, PhD[1]; Carla Lluís-Ganella, MSc[1]; Isaac Subirana, MSc[2,1]; Muntaser D Musameh, MD, PhD[5,6]; Juan Ramon Gonzalez PhD[3,2,4]; Christopher P Nelson PhD[7,8]; Mariano Sentí, MD, PhD[1,7]; The Myocardial Infarction Genetics Consortium†; The Wellcome Trust Case Control Consortium†; Stephen M Schwartz PhD[8]; David Siscovick, MD, MPH[8]; Christopher J O'Donnell MD MPH[9,10]; Olle Melander MD, PhD[11]; Veikko Salomaa MD, PhD[12]; Shaun Purcell PhD[13,14]; David Altshuler, MD, PhD[15,16,17]; Nilesh J Samani, MD, FMedSci[5,6]; Sekar Kathiresan MD[18,15,19]; Roberto Elosua, MD, PhD[1,2].

1 Cardiovascular Epidemiology and Genetics, IMIM. Barcelona, Spain.
2 Epidemiology and Public Health Network (CIBERESP), Barcelona, Spain.
3 Center for Research in Environmental Epidemiology (CREAL), Barcelona, Spain.
4 IMIM (Hospital del Mar Research Institute), Barcelona, Spain.
5 Department of Cardiovascular Sciences, University of Leicester, Leicester, United Kingdom.
6 Leicester NIHR Biomedical Research Unit in Cardiovascular Disease, Glenfield Hospital, Leicester United Kingdom.
7 Pompeu Fabra University. Barcelona, Spain.
8 Cardiovascular Health Research Unit, Departments of Medicine and Epidemiology, University of Washington, Seattle, Washington, USA.
9 National, Heart, Lung, and Blood Institute and Framingham Heart Study, Framingham, Massachusetts, USA.
10 Cardiology Division, Massachusetts General Hospital, Harvard Medical School, Boston, Massachusetts, USA.
11 Department of Clinical Sciences, Hypertension and Cardiovascular Diseases, University Hospital Malmö, Lund University, Malmö, Sweden.
12 Department of Chronic Disease Prevention, National Institute for Health and Welfare, Helsinki, Finland.
13 Psychiatric and Neurodevelopmental Genetics Unit, Massachusetts General Hospital, Boston, Massachusetts, USA.
14 Stanley Center for Psychiatric Research, Broad Institute, Cambridge, Massachusetts, USA.
15 The Broad Institute of MIT and Harvard, Cambridge, Massachusetts, USA.
16 Department of Genetics, Harvard Medical School, Boston, Massachusetts, USA.
17 Department of Molecular Biology, Massachusetts General Hospital, Boston, Massachusetts, USA.
18 Cardiovascular Research Center and Center for Human Genetic Research, Massachusetts General Hospital and Harvard Medical School, Boston, Massachusetts, USA.
19 Department of Medicine, Harvard Medical School, Boston, USA.
† See Appendices for a full list of contributors

CONTENTS Page

Sections, tables and figures in the Supporting Information are indicated by the prefix S (Sx.x, S.Tx and S.Fx, respectively).

Methods *2*

1. Source data from the MIGen discovery and WTCCC validation samples *2*
2. Selection of Risk Factor SNPs and Marginal SNPs *2*
   - 2.1. Source literature for SNP selection *2*
   - 2.2. Definition of risk factor phenotype categories *3*
   - 2.3. Selection of risk factor SNPs *3*
   - 2.4. Selection of marginal SNPs *4*
3. Statistical Analysis *4*
   - 3.1. Tests for association with MI for risk factor SNPs *4*
   - 3.2. Filtering SNP lists to remove redundancy via LD (pruning) *4*
   - 3.3. Statistical tests for gene-gene interaction *4*
   - 3.4. Multiple testing *5*
   - 3.5. Quantile-Quantile plots *6*
   - 3.6. Post-hoc power calculations *6*
   - 3.7. Analysis of Lp(a) variants *8*
   - 3.8. Validation and meta-analysis of top results *9*

Results *10*

4. Risk factor SNP Selection *10*
5. Single locus test for association between risk factor SNPs and MI in the MIGen study. *10*
6. Pair-wise SNP-SNP interaction analysis *10*
7. Adjustment for multiple testing *10*
8. Post-hoc power calculation *11*
9. Analysis of Lp(a) variants *11*

Tables *12*

1. Cardiovascular risk factor SNPs *12*
2. Results for top interactions in MIGen, validation in WTCCC, and meta-analysis. *17*
3. Power Computation *21*

Figures *24*

1. Source literature and process for selection of risk factor SNPs *24*
2. Graphical representation of interaction pairs tested in each Analysis. *25*
3. Computation of significance threshold with adjustment for multiple testing *26*
4. Power computation *28*

Note 1 – Joint case-control/case-only interaction analysis *29*

Note 2 – Logic Regression analysis *32*

Appendix 1 – MIGen investigators *36*

Appendix 2 – WTCCC investigators *37*

References *38*

Methods

1. Source data from the MIGen discovery and WTCCC validation samples

Discovery sample: The Myocardial Infarction Genetics (MIGen) study is a collaborative study whose aim is to explore the genetic basis of myocardial infarction (MI). Our initial study consisted of 2,967 cases of early-onset myocardial infarction (in men ≤50 years old or women ≤60 years old) and 3,075 age- and sex-matched controls free of MI from six international sites in the US (Boston – Massachusetts General Hospital Premature Coronary Artery Disease Study; Seattle - Heart Attack Risk in Puget Sound), Sweden (Malmö Diet and Cancer Study), Finland (FINRISK), Spain (REGICOR) and Italy (ATVB) (see[1] for details). At each site, MI was diagnosed on the basis of autopsy evidence of fatal MI or a combination of chest pain, electrocardiographic evidence of MI, or elevation of one or more cardiac biomarkers (creatine kinase or cardiac troponin). Mean age at the time of MI was 41 years among male cases and 47 years among female cases. All participants were of European ancestry. For these individuals, genotype data were obtained for ~2.55 million single nucleotide polymorphisms, either through direct genotyping (Affymetrix 6.0 GeneChip) or by imputation (MACH 1.0 software[2]), using phased chromosomes from the HapMap CEU sample[1].

Validation sample: Validation of the top results in the discovery sample was performed in a sample of 1,766 cases of coronary artery disease (CAD) and 2,938 controls from the Wellcome Trust Case Control Consortium[3]. CAD cases presented a history of myocardial infarction or coronary revascularization (including coronary bypass surgery or coronary angioplasty) before the age of 60 (see table).

**MI Status**	**N**	**Additional Cardiovascular Phenotype**			**Age**	**Proportion of females**
		Angina	*PCI*	*CABG*	*mean (SD)*	*%*
MI	1280	908	279	561	47,9 (7,1)	19,3
No MI	486	471	176	366	50,5 (6,2)	22,2

PCI, percutaneous coronary intervention; CABG, coronary artery bypass grafting

The control subjects were selected from 2 different studies: a) a British cohort of people born in 1958 (1958 Birth Cohort Controls, 58C); and b) blood donors (UK Blood Services Controls, NBS). Mean age of cases and controls was 48.6 years and 43.7 years, respectively.

Extended details of the methods implemented in these studies are provided in the original manuscripts.

2. Selection of Risk Factor SNPs and Marginal SNPs

2.1. Source literature for SNP selection

To perform interaction analyses for SNPs associated with cardiovascular risk factors, we obtained data from published GWA studies that studied these traits. We identified SNPs of interest by, i) filtering the NHGRI catalogue of GWA studies[4], and ii) mining data from a series of recently published large meta-analyses of GWA studies of cardiovascular risk factors (S.F1).

From the NHGRI catalogue (accessed June 30th, 2010), we filtered the list of reported phenotypes to identify those we considered relevant to cardiovascular disease (S.F1, second column), and identified 48 GWA studies of interest. For subsequent analyses, the accuracy of the all reported associations (p-values, direction of effect, effect allele, etc.) for all SNPs in all relevant articles was verified in the original report, and in cases of discrepancy the data from the original report was used. We also identified eight large meta-analyses of GWA studies of phenotypes of interest that were published after June 30th, 2010 (S.F1, third column). Data for SNPs selected in this process are shown in S.T1.

2.2. Definition of risk factor phenotype categories

We grouped the phenotypes reported in these studies into 11 categories broadly definable as distinct cardiovascular risk factors or cardiovascular endpoints. These were LDL cholesterol (*LDL*), HDL cholesterol (*HDL*), Triglycerides (*TG*), Smoking (*SMK*), Blood Pressure (*BP*), Carbohydrate Metabolism (including Type 2 Diabetes (*T2D*), see below) (*CH*), Obesity/Body Mass (*OB*), Plasma LP(a) levels (*LP(a)*), Concentration of Small LDL Particles (*smallLDL*), and Coronary Artery Disease (*CAD*).

We defined the category *CH* because, in addition to variants associated with overt T2D, we wanted to be able to capture variants that may contribute to cardiovascular risk through association with T2D-related traits, but that may or may not have also been declared as being associated with T2D as a clinical endpoint. Thus, this category contains variants associated with insulin and plasma glucose traits as well as variants associated with overt T2D[5,6]. We used data from a recent GWAS of NMR-based measurements related to lipid quality[7] to test for interaction between variants associated with relative concentrations of small LDL particles and those associated with other CVRFs. The BP category was composed of SNPs associated with either systolic or diastolic blood pressure because in the original reports, most of these SNPs were not observed to have markedly dissimilar effects on these phenotypes.

2.3. Selection of risk factor SNPs

From the studies mentioned above we selected SNPs with a reported p-value of $\leq 5\times10^{-8}$ for association with the phenotype of interest, irrespective of their level of association with other phenotypes in the

case of overlap/pleiotropy. This literature search resulted in a list of 364 SNPs, of which 242 remained after LD pruning (see S3.2). Details of the SNPs included in the pair-wise interaction analysis are given in S.T1. These SNPs were included in the analyses performed in Analyses 1 and 2.

2.4. Selection of marginal SNPs

For Analyses 2 and 3, we used a threshold approach to select SNPs between which interaction testing was to be performed. We selected SNPs that achieved an arbitrary p-value of $\leq 10^{-3}$ (Analysis 2 and 3a) or $\leq 10^{-2}$ (Analysis 3b) for association with MI in the discovery phase of the MIGen study. These lists of SNPs were pruned to remove redundancy through LD (S3.2).

3. Statistical Analysis

All statistical analyses were carried out using packaged or custom functions written in R v2.11 (R Foundation for Statistical Computing, Vienna[8]; packages and functions indicated below by *<package>::<function>*), or using PLINK v1.07[9] where indicated.

3.1. Tests for association with MI for risk factor SNPs

The selection of marginal SNPs was based on the results of the test for single locus association with early-onset MI (age- and sex-matched, with adjustment for ancestry principal components, analyzed using PLINK), as reported previously[1]. See S.T1 for single locus association results for risk factor SNPs.

3.2. Filtering SNP lists to remove redundancy via LD (pruning)

To remove redundancy between SNPs, we applied an LD-based pruning technique implemented in the `--indep-pairwise` function in PLINK. This procedure allowed us to ensure that interaction testing was performed only between mutually independent SNPs, with pair-wise $r^2<0.5$. We also avoided redundancy between Analyses by eliminating from Analyses 2, 3a and 3b any SNPs that had been included in a previous Analysis or that were in LD ($r^2 \geq 0.5$) with any SNPs from a previous Analysis. LD calculations were also performed using PLINK (S.F2).

3.3. Statistical tests for gene-gene interaction

Test A – Model-free case-control test: SNPs were coded as factors with three levels corresponding to their three genotypes. Thus, this test can be thought of as the two-locus equivalent of a single locus

2df genotypic test, in that it assumes no genetic model, but simply searches for differences between cases and controls in the frequencies of the 9 possible compound genotypes (4df; degrees of freedom). In Analyses 1 and 2, we tested for interaction between SNPs by using a likelihood ratio test to compare the fit of a logistic regression model (*stats::glm*) containing a SNP-SNP interaction term to that of an equivalent model lacking this interaction term. These models were adjusted for age, sex and the first two principal components (PC) from an IBS analysis.

MI	~	SNPA + SNPB	+	SNPA X SNPB	+	AGE + SEX + PC1 + PC2
Dichotomous response variable		Main effects (3 categories; 4 parameters)		Interaction terms (4 parameters)		Covariates (incl. IBS principal components)

Test B – Allelic interaction test: Due to the computational requirements of Analysis 3, we performed this analysis using the allelic interaction test implemented in the more efficient `--fast-epistasis` function implemented in PLINK, which uses a joint case-control/case-only strategy to compare a 1df test for correlation between alleles among cases to that among controls[9]. Low MAF for some SNPs leads to low counts in some cells in the genotype collapsing procedure used in this test, such that the test cannot be performed for all pairs; however, this should not lead loss of information because power to detect interaction is very limited for SNPs with low MAF.

3.4. *Multiple testing*

Pair-wise gene-gene interaction testing involves a very large multiple testing burden, requiring appropriate adjustment. However, these tests cannot be considered to be independent because each SNP is involved in multiple tests (N-1 tests for a set of N SNPs), which would make a Bonferroni correction excessively stringent. We approached this problem by estimating the distribution of the most significant p-value in each Analysis under the null hypothesis (simulated by randomizing MI status), which is expected to follow a beta-distribution with parameters determined by the number of tests and the level of correlation between them. To estimate these parameters we computed an approximation to the beta-distribution by taking the most significant p-value from each of large number of permutations of the analysis under H0, and setting the 95th percentile of the resulting beta-distribution as the significance level required to achieve a overall Type I error rate of α=0.05 within each Analysis. *A priori*, the distribution of the minimum p-value for a given set of SNPs is not affected by the type of test used, so we performed the permutations for all Analyses using the more computationally efficient `--fast-epistasis` function in PLINK. The parameters of the beta-

distribution were estimated using 10,000 permutations in Analysis 1. We observed that estimations of these parameters, and consequently of the significance thresholds, were stable after ~200-300 permutations (see Figure 2 in the main manuscript). Therefore, the estimations of these parameters in Analyses 2, 3a and 3b were based on just 1,000, 1,000 and 200 permutations, respectively (See S.F3).

We set the p-value threshold for declaring statistical significance in the validation of top results in the WTCCC as 0.05 with a standard Bonferroni correction for the number of pairs for which we attempted validation in each Analyses (thresholds shown in the results section of the main manuscript).

For the meta-analysis, we set the p-value threshold for declaring statistical significance to be equal to the thresholds used in the discovery analysis of each Analysis (S7).

3.5. Quantile-Quantile plots

To construct a quantile-quantile (QQ) plot in a GWAS setting, the observed test statistics (e.g. χ^2 values) are plotted against their expected distribution under H0. Since the majority of SNPs will not to show any true association with the phenotype under study (in the absence of population stratification), their test statistics will be mutually independent and will follow a parametric distribution (e.g. the χ^2 distribution). However, because of the potential non-independence between pair-wise gene-gene interaction tests, their results may not follow a parametric distribution under H0. To estimate this expected distribution, we used a permutation-based approach similar to that used to compute the threshold for statistical significance in each stage of the interaction analysis (see S3.4).

We performed 1,000 permutations of the analyses under H0 (randomized MI status), and obtained the rank order of all tests within each permutation. Then, for each rank we took the median across all permutations as the expected value for that rank under H0, and plotted this median against the observed value for that rank. The 95% confidence interval of the estimation of the expected distribution was computed by taking the 2.5th and 97.5th percentiles all permutations within each rank. QQ plots for Analysis 1 are provided in Figure 2 in the main manuscript, and for Analyses 2, 3a and 3b in S.F3.

3.6. Post-hoc power calculations

We considered that the power of our analysis to detect interactions was a function of sample size (fixed for this study), an acceptable Type I error rate (α, derived from the beta-distribution), the interaction effect size, the interaction model (e.g. dominant-recessive, etc.), and the frequency of the

compound genotype(s) that carry additional risk. Since the latter three parameters are expected to vary for every pair of SNPs, it is not possible to compute a single value to describe the study's power. Thus, we have dealt with these unknown parameters as follows:

Interaction effect size: Since the true interaction effect size we may expect to find is unknown and will change for every SNP pair, we have expressed the power of the study in terms of the effect size that could be detected with 80% power ($\beta^{0.8}$).

Interaction model: Since the true interaction model is unknown, we computed power to detect interactions under 3 different interaction models: a model with recessive × recessive effects, which is intrinsically the least powerful model because the non-reference group is small; a model with dominant × dominant effects, which is one of the most powerful models because the non-reference group is large, while being very simple because it is driven by the presence of the interacting alleles for each SNP; and a model with additive × additive effects, which is arguably the most biologically plausible for complex diseases. The recessive × recessive and dominant × dominant interaction models mentioned in this paper correspond to those referred by Li and Reich[10] as models M1 (RR), which requires two copies of the interacting allele from both loci to modify disease risk, and M27 (DD), which requires at least one copy of the interacting allele from both loci to modify disease risk. In classical genetics these models are also called 'recessive complementary' and 'dominant complementary' epistasis or 'duplicate dominant' and 'duplicate recessive' epistasis, respectively. The additive × additive model corresponds to that referred to as Model 2 by Marchini *et al.*[11], in which the ln(odds) for disease risk has a baseline value unless both loci have at least one disease-associated allele, after which ln(odds) increases additively within and between genotypes.

Frequency of the risk compound genotype(s): Within the range of MAFs from 0.02 to 0.5, we defined MAF bins of 0.02 (i.e. 0.02≥MAF>0.04, 0.04≥MAF>0.06, etc.; 24 bins for each SNP, giving 24^2=576 bin combinations). For each bin combination, we computed (see below) the mean $\beta^{0.8}$ (effect size detectable with high power) of 10 randomly selected pairs of SNPs whose MAFs fell within these bins.

Power Computation: For each pair of SNPs selected, we computed $\beta^{0.8}$ as follows:

- A model free logistic regression model including a term for interaction SNPs was fit, from which the block of the estimated variance-covariance matrix, V, corresponding to the 4 interaction effects was obtained.
- The 4-component vector corresponding to the values of the 4 interaction terms under the alternative hypothesis, $\vec{\beta}$, was defined under the 3 interaction models as follows:

a. recessive × recessive: $\vec{\vec{\beta}} = \left(0,0,0,\beta^{0.8}\right)^t$

b. dominant × dominant: $\vec{\vec{\beta}} = \left(\beta^{0.8},\beta^{0.8},\beta^{0.8},\beta^{0.8}\right)^t$

c. additive × additive: $\vec{\vec{\beta}} = \left(\beta^{0.8},2\beta^{0.8},2\beta^{0.8},4\beta^{0.8}\right)^t$

- Finally, $\beta^{0.8}$ was obtained by solving the following equation

$$0.8 = P\left(\chi_4^2(\gamma) > c\right),$$

where *c* is the 95th percentile of χ_4^2, $\chi_4^2(\gamma)$ is a chi-squared variable with 4 degrees of freedom and non-centrality parameter $\gamma = \vec{\vec{\beta}}^t V^{-1} \vec{\vec{\beta}}$.

In Analyses 1, 2 and 3a, data on variance, V, for each of a series of SNP pairs, was obtained from the actual interaction tests performed in that Analysis. For Analysis 3b, we assumed that variances would be similar to those computed for Analysis 3a, so we computed power from these variances, but using the Type I error rate (α) computed for Analysis 3b.

The results of these power calculations are shown in S.T3 and S.F4.

3.7. Analysis of Lp(a) variants

Recent studies have highlighted the potential relevance of lipoprotein(a) (Lp(a), encoded by the *LPA* gene) as a cardiovascular risk factor (see[12]). Clarke *et al.*[13] observed that two SNPs in LPA, rs3798220 and rs10455872, were strongly associated with risk of CAD, and noted that rs3798220 was in strong LD (r^2=0.86) with a four-SNP haplotype previously reported by Trégouët *et al*[14] as also being associated with CAD.

While neither of these SNPs was available in the MIGen genotype data, the four SNPs (rs2048327, rs3127599, rs7767084 and rs10755578) that comprised the Trégouët haplotypes were available. To attempt to capture the CAD risk-associated variation in LPA we re-constructed the Trégouët haplotypes in the MIGen sample (S.9), verified the association between these haplotypes and risk of MI, and then tested for interaction between these haplotypes and the CVRF SNPs.

We tested for direct association between MI and these haplotypes using the *haplo.stats::haplo.glm* function[15] to fit a logistic regression model of MI risk on haplotype effects, while accounting for ambiguity in the assignment of haplotypes; this model was adjusted for age, sex and IBS principal components.

To test for evidence of interaction between these haplotypes and the 242 CVRF SNPs as a predictor of MI risk, we used a likelihood ratio test to compare the fit of a regression model (fit using

haplo.stats::haplo.glm) containing an haplotype-SNP interaction term to an equivalent model lacking this term, again with adjustment for age, sex and IBS principal components. We used a Bonferroni correction to set the threshold for declaring statistical significance. The results of the test for association between these haplotypes and MI risk, and those for interaction between the haplotypes and CVRF SNPs are shown in S.9.

3.8. Validation and meta-analysis of top results

Validation. We selected all SNP pairs with p-values for interaction in the MIGen discovery sample within 3 orders of magnitude of the significance threshold within each Analysis: $p \leq 1.51 \times 10^{-3}$, $p \leq 3.13 \times 10^{-4}$, $p \leq 2.93 \times 10^{-4}$ and $p \leq 3.57 \times 10^{-6}$ for Analyses 1, 2 and 3a and 3b, respectively. Using the same interaction testing procedure (reproduced faithfully in the discovery and validation samples using a standardized R-script to perform data formatting, quality control and interaction testing), we validated these top results in a sample of CAD cases and controls from the WTCCC (S1). [Note that the top results in Analyses 3a and 3b, which were initially computed using Test B in the MIGen sample, were reproduced using Test A in the MIGen sample for the purpose of including them in the meta-analysis.]

Meta-analysis. We performed a fixed effects meta-analysis by pooling the β-coefficients of the interaction terms from the models for each study, weighted by the inverse of their variances, as follows:

For each interaction term (*j*=1, 2, 3, 4), the pooled β-coefficient of the interaction term was computed as $\hat{\beta}_{pooled}(j) = \frac{\hat{\beta}_1(j) \cdot w_1(j) + \hat{\beta}_2(j) \cdot w_2(j)}{w_1(j) + w_2(j)}$, where $\hat{\beta}_1$ and $\hat{\beta}_2$ were the β-coefficients and w_1 and w_2 the weights of the interaction terms for the MIGen and WTCCC samples, respectively. The variance-covariance matrix of the $\hat{\beta}_{pooled}$ vector was computed as $V_{pooled} = \left[V_1^{-1} + V_2^{-1}\right]^{-1}$, where, V_1 and V_2 are the variance-covariance matrices of beta-coefficients of the interaction terms for the MIGen and WTCCC samples, respectively. The vector weights, w_1 and w_2 were computed as $w_1(j) = 1/V_1(j, j)$ and $w_2(j) = 1/V_2(j, j)$, where $M(j, j)$ is the *j*-th element of the diagonal of the matrix *M*. The test for interaction consisted of testing whether or not all interaction terms are equal to zero [H0: $\beta_{pooled} = (0,0,0,0)^t$; H1: $\beta_{pooled} \neq (0,0,0,0)^t$] by computing the following statistic, which corresponds to a Wald test: $\chi^2 = \hat{\beta}_{pooled}^t V_{pooled}^{-1} \hat{\beta}_{pooled}$. This test statistic follows a χ^2 distribution with 4 degrees of freedom under the null hypothesis.

Results

4. Risk factor SNP Selection

We selected 242 independent SNPs for interaction analysis on the basis of their association with CV risk factors or clinical endpoints. dbSNP rs# identifiers, reported phenotype, local gene(s), p-value for association with MI in the MIGen study, minor allele frequency in MIGen controls, and references of studies that discovered or verified the association are shown in S.T1. The literature sources and process used to select these SNPs are described in S.F1.

5. Single locus test for association between risk factor SNPs and MI in the MIGen study.

A full list of the results of the single locus test for association between the CVRF SNPs and MI is given in S.T1.

6. Pair-wise SNP-SNP interaction analysis

Results for SNP pairs that showed a p-value for interaction within 3 orders of magnitude of the threshold for statistical significance in Analyses 1, 2, 3a and 3b are shown in S.T2. These SNP pairs were brought forward for validation in the WTCCC sample; results for interaction in WTCCC and for meta-analysis of both studies are also shown in S.T2.

7. Adjustment for multiple testing

Figure 2 in the main manuscript and S.F3 show the distribution of the minimum p-values for a large number of permutations under the null hypothesis, as well as the corresponding beta distribution from which the threshold for declaring statistical significance was computed in each Analysis. The following table compares Bonferroni corrected significance levels (α=0.05/number of tests) in each Analysis to the empirically derived thresholds:

	Empirical Threshold	Bonferroni Threshold	Number of tests
**Analysis 1**	1.51×10^{-6}	1.71×10^{-6}	29,161
**Analysis 2**	3.13×10^{-7}	3.21×10^{-7}	155,606
**Analysis 3a**	2.93×10^{-7}	2.42×10^{-7}	201,537
**Analysis 3b**	3.57×10^{-9}	2.75×10^{-9}	17,470,706

8. Post-hoc power calculation

For a range of MAFs and interaction models, we computed the interaction effect size that our study could detect with 80% power (S.T3, S.F4). These calculations give a two dimensional array of effect sizes (one dimension for each SNP) for three interaction models, recessive × recessive, dominant × dominant and additive × additive (S.T3, S.F4).

9. Analysis of Lp(a) variants

Analysis of direct association between Trégouët haplotypes and MI risk. We tested for association between haplotypes reported by Trégouët *et al*.[14] and risk of MI in the MIGen study and found similar results, with the CCTC (p=0.000077; OR [95%CI]=1.71 [1.31,2.22]) and CTTG (p=0.0278; OR=1.14 [1.01,1.28]) haplotypes showing similar effects on risk (more common in cases) to those previously reported, and in the same direction.

				Frequency in MIGen		odds ratio	95% CI	association p-value
				controls	cases			
T	C	T	C	0.503	0.495		*reference haplotype*	
C	C	C	G	0.148	0.141	0.97	0.87,1.08	0.610541
C	C	T	C	0.019	0.031	1.71	1.31,2.22	0.000077
C	C	T	G	0.021	0.018	0.91	0.70,1.19	0.481655
C	T	T	G	0.115	0.125	1.14	1.01,1.28	0.02776
T	T	T	C	0.018	0.016	0.93	0.69,1.25	0.63506
T	T	T	G	0.158	0.157	1.00	0.90,1.11	0.991476

Trégouët haplotypes are shown in columns 1 to 4, with SNPs in the following order: rs2048327, rs3127599, rs7767084, rs10755578

Analysis of interaction between Trégouët haplotypes and CVRF SNPs as a predictor of MI risk. We observed no significant evidence for interaction between the Trégouët haplotypes and 240 of the 242 CVRF SNPs, after correction for multiple testing (significance threshold, p=0.00021). The *haplo.glm* regression models containing terms for interaction between the Trégouët haplotypes and rs1800961 and rs6919346 failed to converge. rs1800961 lies at the HNF4A locus and was previously reported to be associated with total and HDL cholesterol levels[16,17]. rs6919346 lies within LPA and was reported by Ober *et al*.[18] to be associated with plasma Lp(a) levels. The most significant p-value for interaction was observed for rs2068888 (p=0.0039), which lies in *CYP26A1* and was previously reported to be associated with plasma triglyceride levels (S.T1).

Tables

Table 1: Cardiovascular risk factor SNPs. Details of the SNPs associated with cardiovascular risk factors (CVRF) and clinical endpoints that were selected for interaction analysis in this study. The following data are shown for each of the 242 SNPs: the reported phenotype(s); nearby gene(s), if reported; p-value for association with MI in the MIGen study; MAF in MIGen controls; references for studies that discovered or replicated the association.

SNP	Chr	Reported Phenotype	Nearby Gene, if reported	p-value for MI in MIGen[a]	MAF in MIGen controls	Reference
rs1333049	9	Coronary disease	Intergenic; CDKN2A; CDKN2B	3.42e-07	0.483	[19,3]
rs6725887	2	Myocardial infarction (early onset)	WDR12	8.55e-05	0.126	[16]
rs1121980	16	Body mass index	FTO	0.00012	0.437	[20]
rs17465637	1	Myocardial infarction (early onset)	MIA3	0.00015	0.293	[16]
rs1746048	10	Myocardial infarction (early onset)	CXCL12	0.000161	0.173	[16]
rs1122608	19	Myocardial infarction (early onset)	LDLR	0.000172	0.264	[16]
rs12526453	6	Myocardial infarction (early onset)	PHACTR1	0.00046	0.362	[16]
rs2000999	16	TC; LDL		0.000726	0.208	[17]
rs9982601	21	Myocardial infarction (early onset)	SLC5A3; MRPS6; KCNE2	0.000782	0.128	[16]
rs964184	11	HDL cholesterol; Triglycerides; TC; LDL; HDL; TG; CAD	APOA1; APOC3; APOA4; APOA5; ZNF259; APOA5-A4-C3-A1	0.00122	0.152	[21,17,22]
rs10423928	19	Two-hour glucose challenge; CH	GIPR	0.0014	0.19	[5,6]
rs7205804	16	TG		0.00148	0.471	[17]
rs2521501	15	BP		0.00153	0.333	[23]
rs3184504	12	Diastolic blood pressure; Systolic blood pressure	SH2B3	0.00157	0.483	[24]
rs649129	9	LDL		0.00295	0.231	[17]
rs7350481	11	Hematological and biochemical traits	APO-A cluster	0.00322	0.079	[25]
rs12779790	10	Type 2 diabetes	CDC123; CAMK1D	0.00357	0.178	[26]
rs2814944	6	HDL		0.0038	0.145	[17]
rs6511720	19	LDL cholesterol; TC; LDL	LDLR	0.0047	0.104	[16,27,17]
rs13139571	4	BP		0.00575	0.273	[23]
rs16948048	17	Diastolic blood pressure	ZNF652; PHB	0.0076	0.368	[28]
rs6544713	2	LDL cholesterol	ABCG8	0.0106	0.334	[16]
rs2844479	6	Weight	AIF1; NCR3	0.0109	0.43	[29]
rs2967605	19	HDL cholesterol	ANGPTL4	0.0196	0.169	[16]
rs3177928	6	TC; LDL		0.0209	0.135	[17]
rs11206510	1	LDL cholesterol; Myocardial infarction (early onset)	PCSK9	0.0212	0.196	[21,16,27]
rs7593730	2	Type 2 diabetes	RBMS1; ITGB6	0.0221	0.219	[30]
rs17114036	1	CAD	PPAP2B	0.0233	0.109	[22]
rs4773144	13	CAD	COL4A1; COL4A2	0.0247	0.459	[22]
rs1532085	15	HDL cholesterol; TC; HDL	LIPC	0.0259	0.373	[31,32,17]
rs17584499	9	Type 2 diabetes	PTPRD	0.0277	0.181	[33]
rs12970134	18	Body mass index; Weight; Waist circumference and related phenotypes	MC4R	0.0303	0.26	[29,34]
rs16998073	4	Diastolic blood pressure	FGF5; PRDM8; c4orf22	0.032	0.207	[28]
rs1004467	10	Systolic blood pressure	CYP17A1	0.033	0.104	[24]
rs2412710	15	TG		0.034	0.029	[17]

rs261342	15	TG		0.0442	0.205	[17]
rs11556924	7	CAD	ZC3HC1	0.0464	0.353	[22]
rs181362	22	HDL		0.0535	0.196	[17]
rs2650000	12	LDL cholesterol	HNF1A	0.0582	0.355	[16]
rs2072183	7	TC		0.0583	0.209	[17]
rs4846914	1	HDL cholesterol; Triglycerides; HDL	GALNT2	0.0583	0.404	[21,16,17]
rs17321515	8	Triglycerides	TRIB1	0.0585	0.459	[21,27]
rs1689800	1	HDL		0.0618	0.383	[17]
rs17609940	6	CAD	ANKS1A	0.063	0.144	[22]
rs7957197	12	T2D	HNF1A	0.0648	0.208	[35]
rs12670798	7	LDL cholesterol; LDL	DH11	0.0692	0.195	[31,17]
rs6922269	6	Coronary disease	MTHFD1L	0.0702	0.248	[19]
rs599839	1	LDL cholesterol; Coronary disease	CELSR2; PSRC1; SORT1	0.0777	0.213	[36,27,19]
rs4420638	19	LDL cholesterol; TC; LDL; HDL	APOE; APOC1; APOC4; APOC2	0.0814	0.156	[21,16,36,27,17]
rs6474412	8	Smoking behavior; SMK	CHRNB3; CHR6	0.0816	0.227	[37]
rs419076	3	BP		0.0843	0.491	[23]
rs9818870	3	Coronary artery disease	MRAS	0.0873	0.137	[38]
rs6548238	2	Body mass index	TMEM18	0.0902	0.194	[39]
rs2929282	15	TG		0.0904	0.059	[17]
rs1501908	5	LDL cholesterol	TIMD4; HAVCR1	0.0953	0.39	[16]
rs7134375	12	HDL		0.0973	0.395	[17]
rs1129555	10	LDL		0.0978	0.325	[17]
rs1564348	6	TC; LDL		0.0979	0.155	[17]
rs1013442	11	SMK		0.0981	0.271	[40]
rs10850411	12	BP		0.103	0.32	[23]
rs16969968	15	SMK		0.106	0.388	[40]
rs10946398	6	Type 2 diabetes	CDKAL1	0.107	0.306	[41]
rs4373814	10	BP		0.111	0.441	[23]
rs2652834	15	HDL		0.112	0.209	[17]
rs4082919	17	HDL		0.112	0.49	[17]
rs46522	17	CAD	UBE2Z; GIP; ATP5G1; SNF8	0.112	0.472	[22]
rs2479409	1	TC; LDL		0.113	0.337	[17]
rs4607103	3	Type 2 diabetes	ADAMTS9	0.116	0.292	[26]
rs1424233	16	Obesity	MAF	0.117	0.49	[42]
rs2068888	10	TG		0.124	0.484	[17]
rs7395662	11	HDL cholesterol	MADD; FOLH1	0.126	0.37	[31]
rs10938397	4	Body mass index	GNPDA2	0.128	0.431	[27]
rs28927680	11	Triglycerides	APOA1; APOC3; APOA4; APOA5; ZNF259; BUD13	0.128	0.074	[21]
rs2902941	20	LDL		0.143	0.303	[17]
rs1552224	11	T2D	CENTD2	0.145	0.13	[35]
rs3846662	5	LDL cholesterol	HMGCR	0.151	0.44	[31]
rs16942887	16	HDL		0.153	0.119	[17]
rs7941030	11	TC		0.159	0.358	[17]
rs386000	19	HDL		0.162	0.155	[17]
rs2247056	6	TG		0.169	0.168	[17]
rs4810479	20	TG		0.171	0.232	[17]
rs3136441	11	HDL		0.18	0.131	[17]
rs3742207	13	Arterial stiffness	COL4A1	0.183	0.353	[43]
rs2814982	6	TC		0.184	0.08	[17]
rs6450176	5	HDL		0.186	0.288	[17]
rs7206971	17	TC		0.192	0.483	[17]
rs805303	6	BP		0.205	0.346	[23]
rs2877716	3	Two-hour glucose challenge; CH	ADCY5	0.21	0.214	[5,6]
rs7120118	11	HDL cholesterol	NR1H3	0.21	0.251	[32]
rs5756931	22	TG		0.213	0.404	[17]

rs13082711	3	BP		0.215	0.215	[23]
rs6759321	2	TC		0.216	0.491	[17]
rs1515100	2	HDL		0.231	0.374	[17]
rs12310367	12	TG		0.235	0.365	[17]
rs7396835	11	Quantitative traits	Intergenic	0.236	0.156	[44]
rs11084753	19	Body mass index	KCTD15	0.242	0.351	[27]
rs217386	7	LDL		0.247	0.453	[17]
rs987237	6	Adiposity	TFAP2B	0.247	0.17	[45]
rs11220462	11	LDL		0.259	0.132	[17]
rs10195252	2	TG		0.263	0.394	[17]
rs243021	2	T2D	BCL11A	0.263	0.458	[35]
rs12946454	17	Systolic blood pressure	PLCD3; ACBD4; HEXIM1; HEXIM2	0.291	0.269	[28]
rs11136341	8	TC; LDL		0.297	0.412	[17]
rs2384550	12	Diastolic blood pressure	TBX3; TBX5	0.306	0.371	[24]
rs17216525	19	Triglycerides	NCAN; CILP2; PBX4	0.316	0.075	[16]
rs925946	11	Body mass index; Weight	BDNF	0.318	0.259	[29]
rs11953630	5	BP		0.319	0.399	[23]
rs7255436	19	HDL		0.325	0.469	[17]
rs174570	11	LDL cholesterol	FADS2; FADS3	0.333	0.117	[31]
rs737337	19	HDL		0.335	0.085	[17]
rs581080	9	TC		0.337	0.205	[17]
rs255049	16	HDL cholesterol	LCAT	0.345	0.211	[32]
rs11776767	8	TG		0.354	0.381	[17]
rs1367117	2	TC; LDL		0.355	0.288	[17]
rs174601	11	HDL		0.357	0.341	[17]
rs4148008	17	HDL		0.357	0.337	[17]
rs3096277	16	Blood pressure	CDH13	0.362	0.195	[24]
rs17608766	17	BP		0.367	0.144	[23]
rs2126259	8	TC; LDL		0.372	0.089	[17]
rs7961581	12	Type 2 diabetes	TSPAN8; LGR5	0.372	0.307	[26]
rs6754295	2	HDL cholesterol; Triglycerides	APOB	0.374	0.256	[31]
rs326	8	Triglycerides	LPL; C8orf35; SLC18A1	0.376	0.327	[46]
rs12130333	1	Triglycerides	ANGPTL3; DOCK7; ATG4C	0.379	0.175	[21]
rs645040	3	TG		0.382	0.226	[17]
rs4689388	4	Type 2 diabetes and other traits	WFS1; PPP2R2C	0.384	0.393	[47]
rs391300	17	Type 2 diabetes	SRR	0.387	0.382	[33]
rs2254287	6	LDL cholesterol	B3GALT4	0.389	0.435	[27]
rs7034200	9	Fasting glucose-related traits; CH	GLIS3	0.39	0.486	[6]
rs1961456	8	TC		0.397	0.321	[17]
rs2568958	1	Body mass index; Weight	NEGR1	0.398	0.336	[29]
rs6919346	6	Plasma Lp (a) levels	LPA	0.4	0.19	[18]
rs7498665	16	Body mass index; Weight	SH2B1; ATP2A1	0.404	0.324	[29,39]
rs442177	4	TG		0.409	0.395	[17]
rs10761731	10	TG		0.412	0.428	[17]
rs1084651	6	HDL		0.415	0.134	[17]
rs864745	7	Type 2 diabetes	JAZF1	0.415	0.491	[26]
rs7811265	7	TG		0.418	0.162	[17]
rs10832963	11	TC		0.419	0.284	[17]
rs1800961	20	HDL cholesterol; TC; HDL	HNF4A	0.421	0.025	[16,17]
rs3905000	9	HDL cholesterol	ABCA1	0.422	0.145	[31]
rs11649653	16	TG		0.431	0.428	[17]
rs3825807	15	CAD	ADAMTS7	0.44	0.433	[22]
rs7647305	3	Body mass index; Weight	SFRS10; ETV5; DGKG	0.44	0.204	[29]
rs514230	1	TC; LDL		0.442	0.468	[17]
rs12190287	6	CAD	TCF21	0.444	0.368	[22]
rs2895811	14	CAD	HHIPL1	0.452	0.399	[22]

rs1167998	1	Triglycerides	DOCK7	0.462	0.307	[31]
rs340874	1	Fasting glucose-related traits; CH	PROX1	0.465	0.489	[6]
rs3741414	12	HDL		0.467	0.193	[17]
rs11558471	8	Fasting glucose-related traits	SLC30A8	0.47	0.282	[6]
rs1799945	6	BP		0.479	0.157	[23]
rs1329650	10	Smoking behavior; SMK	LOC100188947	0.48	0.259	[40]
rs4759375	12	HDL		0.49	0.061	[17]
rs10892151	11	Triglycerides	APOA1; APOC3; APOA4; APOA5; DSCAML1	0.511	0.032	[48]
rs2075292	11	Triglycerides	APOA1; KIAA0999; LOC645044	0.52	0.133	[46]
rs17367504	1	Systolic blood pressure	MTHFR; NPPA; CLCN6; NPPB; AGTRAP	0.523	0.129	[28]
rs13107325	4	HDL; BP		0.525	0.083	[17,23]
rs3757354	6	TC; LDL		0.529	0.196	[17]
rs11920090	3	Fasting glucose-related traits; CH	SLC2A2	0.532	0.151	[6]
rs231362	11	T2D	KCNQ1	0.533	0.486	[35]
rs10885122	10	Fasting glucose-related traits; CH	ADRA2A	0.536	0.129	[6]
rs2293889	8	HDL		0.552	0.398	[17]
rs4939883	18	HDL cholesterol; smallLDL	LIPG	0.554	0.159	[31,16,7]
rs515135	2	LDL cholesterol	APOB	0.555	0.186	[16]
rs11153594	6	LDL		0.558	0.411	[17]
rs4149268	9	HDL cholesterol	ABCA1	0.578	0.392	[27]
rs643531	9	HDL		0.596	0.138	[17]
rs2290159	3	TC		0.607	0.215	[17]
rs10146997	14	Waist circumference	NRXN3	0.611	0.188	[49]
rs2332328	14	LDL		0.616	0.484	[17]
rs12936587	17	CAD	RASD1; SMCR3; PEMT	0.618	0.43	[22]
rs11634397	15	T2D	ZFAND6	0.619	0.341	[35]
rs4731702	7	HDL		0.621	0.462	[17]
rs1030431	8	TC; LDL		0.624	0.36	[17]
rs7944584	11	Fasting glucose-related traits; CH	MADD	0.625	0.317	[6]
rs12328675	2	HDL		0.63	0.139	[17]
rs10830963	11	Fasting glucose-related traits; Fasting plasma glucose; CH	MTNR1B	0.633	0.281	[6,50]
rs7129220	11	BP		0.638	0.116	[23]
rs1111875	10	Type 2 diabetes	HHEX	0.641	0.393	[51,52,53]
rs2166706	11	Fasting plasma glucose	MTNR1B	0.641	0.42	[54]
rs11014166	10	Diastolic blood pressure	CACNB2	0.643	0.375	[24]
rs932764	10	BP		0.647	0.415	[23]
rs2681492	12	Systolic blood pressure	ATP2B1	0.65	0.198	[24]
rs7826222	8	Adiposity	MSRA	0.658	0.192	[45]
rs7578597	2	Type 2 diabetes	THADA	0.667	0.107	[26]
rs2923084	11	HDL		0.67	0.192	[17]
rs1327235	20	BP		0.68	0.458	[23]
rs6102059	20	LDL cholesterol	MAFB	0.694	0.27	[16]
rs2277862	20	TC		0.697	0.18	[17]
rs2807834	1	TC; LDL		0.698	0.322	[17]
rs3774372	3	BP		0.701	0.204	[23]
rs2191349	7	Fasting glucose-related traits; CH	DGKB; TMEM195	0.705	0.451	[6]
rs1173771	5	BP		0.709	0.394	[23]
rs11071657	15	Fasting glucose-related traits; CH	C2CD4B	0.713	0.363	[6]
rs492602	19	TC		0.713	0.479	[17]
rs3733829	19	Smoking behavior; SMK	CYP2A6; EGLN2	0.714	0.368	[40]
rs10096633	8	Triglycerides; Other metabolic traits	LPL	0.716	0.156	[31,32]
rs7515577	1	TC		0.716	0.189	[17]
rs4105144	19	Smoking behavior; SMK	CYP2A6; RAB4D	0.717	0.284	[37]

rs381815	11	Systolic blood pressure	PLEKHA7	0.726	0.252	[24]
rs10923931	1	Type 2 diabetes	NOTCH2; ADAM30	0.735	0.098	[26]
rs7134594	12	HDL		0.736	0.442	[17]
rs1260326	2	Triglycerides; Other metabolic traits; Two-hour glucose challenge; CH; TC; TG; largeHDL.	GCKR	0.743	0.474	[16,32,5,6,17,7]
rs9989419	16	HDL cholesterol	CETP	0.744	0.404	[55,27]
rs4506565	10	Type 2 diabetes; Fasting glucose-related traits	TCF7L2	0.747	0.356	[3,6]
rs560887	2	Fasting glucose-related traits; Other metabolic traits; Fasting plasma glucose; CH	G6PC2; ABCB11	0.749	0.281	[6,32,50,56]
rs3025343	9	Smoking behavior; SMK	DBH	0.754	0.101	[40]
rs7832552	8	Body mass (lean)	TRHR	0.755	0.278	[57]
rs909802	20	LDL		0.759	0.469	[17]
rs5215	11	Type 2 diabetes	KCNJ11	0.761	0.361	[41]
rs896854	8	T2D	TP53INP1	0.765	0.474	[35]
rs17271305	15	CH		0.768	0.368	[6]
rs2605100	1	Adiposity	LYPLAL1	0.769	0.272	[45]
rs2737229	8	TC		0.77	0.285	[17]
rs881844	17	HDL		0.771	0.328	[17]
rs2925979	16	HDL		0.772	0.298	[17]
rs9686661	5	TG		0.791	0.217	[17]
rs838880	12	HDL		0.8	0.323	[17]
rs11605924	11	Fasting glucose-related traits; CH	CRY2	0.814	0.468	[6]
rs2237892	11	Type 2 diabetes	KCNQ1	0.815	0.054	[51,58]
rs4607517	7	Fasting glucose-related traits; Fasting plasma glucose; CH	GCK	0.823	0.191	[6,50]
rs633185	11	BP		0.841	0.27	[23]
rs1800562	6	TC; LDL		0.857	0.037	[17]
rs693	2	LDL cholesterol	APOB	0.861	0.461	[31,32,21]
rs35767	12	Fasting glucose-related traits; Fasting insulin-related traits; CH	IGF1	0.865	0.181	[6]
rs6015450	20	BP		0.868	0.104	[23]
rs2932538	1	BP		0.886	0.275	[23]
rs7225700	17	LDL		0.888	0.336	[17]
rs8042680	15	T2D	PRC1	0.896	0.361	[35]
rs6495122	15	Diastolic blood pressure	CSK; ULK3	0.903	0.493	[24]
rs10913469	1	Weight	SEC16B; RASAL2	0.914	0.171	[29]
rs6499640	16	Body mass index; Weight	FTO	0.915	0.389	[29]
rs6769511	3	Type 2 diabetes	IGF2BP2	0.918	0.313	[59]
rs12027135	1	TC; LDL		0.93	0.494	[17]
rs4660293	1	HDL		0.934	0.225	[17]
rs2383208	9	Type 2 diabetes	CDKN2A; CDKN2B	0.935	0.193	[51]
rs1530440	10	Diastolic blood pressure	c10orf107; TMEM26; RTKN2; RHOBTB1; ARID5B	0.944	0.187	[28]
rs1531343	12	T2D	HMGA2	0.945	0.141	[35]
rs10838738	11	Body mass index	MTCH2	0.953	0.339	[39]
rs13292136	9	T2D	CHCHD9	0.955	0.053	[35]
rs605066	6	HDL		0.961	0.414	[17]
rs7819412	8	Triglycerides	XKR6; AMAC1L2	0.967	0.492	[16]

[a] p-value for association with MI in the MIGen study, with adjustment for age, sex and genetic principal components

Table 2: Results for top interactions in MIGen, validation in WTCCC, and meta-analysis. SNP pairs with p-value for interaction in the MIGen study within 3 orders of magnitude of the significance threshold in each Analysis are shown in order of decreasing significance. Results for Analyses 2, 3a and 3b are shown on the following three pages.

Analysis 1

SNP1	SNP2	MAF SNP1[a]	MAF SNP2[a]	Discovery Phenotype SNP1	Discovery Phenotype SNP2	MIGen interaction p-value	WTCCC interaction p-value	Meta-analysis interaction p-value
rs2072183	rs1013442	0.209	0.271	TC; LDL	SMK	5.54E-06	8.39E-01	2.08E-02
rs11220462	rs5756931	0.132	0.404	TC; LDL	TG	8.32E-06	3.99E-01	7.25E-04
rs7120118	rs4810479	0.251	0.232	HDL	TG; HDL	4.75E-05	5.77E-01	7.55E-03
rs2737229	rs381815	0.285	0.252	TC	BP	6.83E-05	8.62E-02	2.78E-04
rs3774372	rs2923084	0.204	0.192	BP	HDL	8.77E-05	9.36E-01	9.91E-03
rs1800562	rs7350481	0.037	0.079	TC; LDL	TG	1.22E-04	9.00E-01	3.83E-02
rs11776767	rs10146997	0.381	0.188	TG	OB	1.67E-04	9.12E-02	1.85E-01
rs3177928	rs925946	0.135	0.259	TC; LDL	OB	1.72E-04	4.07E-01	2.75E-03
rs12190287	rs1030431	0.368	0.360	MI/CAD	TC; LDL	2.34E-04	5.05E-01	2.18E-04
rs645040	rs12190287	0.226	0.368	TG	CAD	2.85E-04	4.13E-03	1.37E-01
rs2126259	rs1129555	0.089	0.325	TC; LDL; HDL	TC; LDL	2.88E-04	4.28E-01	9.06E-02
rs11558471	rs231362	0.282	0.486	CH	CH	3.01E-04	2.54E-01	2.10E-02
rs2807834	rs9982601	0.322	0.128	TC; LDL	MI/CAD	3.05E-04	2.37E-01	6.98E-04
rs6759321	rs7961581	0.491	0.307	TC	CH	3.08E-04	8.04E-01	3.06E-03
rs6474412	rs1746048	0.227	0.173	SMK	MI/CAD	3.23E-04	1.87E-02	5.81E-02
rs10892151	rs6102059	0.032	0.270	TG	LDL	3.55E-04	9.27E-01	1.92E-02
rs1329650	rs16969968	0.259	0.388	SMK	SMK	5.56E-04	4.73E-01	2.08E-03
rs2247056	rs11556924	0.168	0.353	TG	MI/CAD	5.85E-04	3.65E-01	8.09E-03
rs693	rs2254287	0.461	0.435	LDL	LDL	5.94E-04	4.27E-01	3.39E-02
rs7578597	rs11953630	0.107	0.399	CH	BP	6.11E-04	5.23E-01	6.86E-02
rs12027135	rs11920090	0.494	0.151	TC; LDL	CH	6.12E-04	5.10E-02	9.70E-03
rs599839	rs7129220	0.213	0.116	LDL; MI/CAD	BP	6.60E-04	2.27E-02	1.23E-01
rs10096633	rs1121980	0.156	0.437	TG	OB	6.80E-04	5.16E-01	6.96E-02
rs4607103	rs6919346	0.292	0.190	CH	LP(a)	7.18E-04	1.70E-02	1.49E-05
rs13082711	rs7255436	0.215	0.469	BP	HDL	7.24E-04	6.79E-01	8.63E-02
rs7832552	rs4939883	0.278	0.159	OB	HDL; smallLDL	7.51E-04	3.18E-01	7.49E-03
rs2650000	rs12946454	0.355	0.269	LDL	BP	7.53E-04	4.17E-01	4.93E-03
rs7350481	rs12946454	0.079	0.269	TG	BP	7.92E-04	6.45E-01	1.42E-02
rs16998073	rs2166706	0.207	0.420	BP	CH	8.08E-04	9.71E-01	3.79E-02
rs9818870	rs6450176	0.137	0.288	MI/CAD	HDL	8.14E-04	5.38E-01	5.34E-03
rs11220462	rs6495122	0.132	0.493	TC; LDL	BP	8.31E-04	5.20E-01	4.46E-02
rs7826222	rs4810479	0.192	0.232	OB	TG; HDL	8.87E-04	--	--
rs391300	rs2277862	0.382	0.180	CH	TC	8.93E-04	2.67E-01	4.95E-04
rs514230	rs881844	0.468	0.328	TC; LDL	HDL	8.98E-04	4.36E-01	5.83E-03
rs1333049	rs11071657	0.483	0.363	MI/CAD	CH	9.18E-04	1.60E-01	1.02E-02
rs46522	rs1327235	0.472	0.458	MI/CAD	BP	9.29E-04	6.59E-01	5.36E-03
rs11014166	rs1746048	0.375	0.173	BP	MI/CAD	9.31E-04	6.44E-01	9.06E-03
rs2075292	rs2412710	0.133	0.029	TG	TG	9.50E-04	8.13E-02	1.48E-01
rs605066	rs6495122	0.414	0.493	HDL	BP	9.95E-04	8.13E-02	9.39E-02
rs13292136	rs909802	0.053	0.469	CH	TC; LDL	9.98E-04	3.00E-01	--[b]
rs3905000	rs10761731	0.145	0.428	HDL	TG	1.02E-03	6.14E-01	2.54E-03
rs2844479	rs12779790	0.430	0.178	OB	CH	1.06E-03	2.81E-01	1.95E-02
rs4846914	rs1800961	0.404	0.025	HDL; TG	TC; HDL	1.10E-03	2.35E-01	3.32E-02
rs605066	rs1084651	0.414	0.134	HDL	HDL	1.12E-03	5.76E-01	2.49E-03
rs1367117	rs6544713	0.288	0.334	TC; LDL	LDL	1.15E-03	1.31E-01	2.40E-04
rs7350481	rs2902941	0.079	0.303	TG	TC; LDL	1.17E-03	4.77E-01	2.81E-02
rs1129555	rs4506565	0.325	0.356	TC; LDL	CH	1.20E-03	3.92E-01	9.80E-02
rs987237	rs28927680	0.170	0.074	OB	TG	1.31E-03	3.31E-02	1.44E-02

[a] Minor allele frequency in MIGen controls

[b] Data were available for both SNPs in this pair, but the meta-analysis model returned an unreliable result due to extreme variance in for some of the interaction terms

Analysis 2

SNP1	SNP2	MAF SNP1[a]	MAF SNP2[a]	p-value for MI, SNP1[c]	p-value for MI, SNP2[c]	MIGen interaction p-value	WTCCC interaction p-value	Meta-analysis interaction p-value
rs3136441	rs9990208	0.123	0.091	1.80E-01	1.20E-04	9.48E-07	8.36E-02	1.52E-02
rs3733829	rs7141502	0.368	0.333	7.14E-01	9.68E-04	9.78E-06	1.24E-01	1.65E-03
rs2293889	rs4076319	0.398	0.171	5.52E-01	7.26E-04	9.83E-06	1.67E-01	4.50E-05
rs12328675	rs12899875	0.137	0.073	6.30E-01	5.39E-04	1.21E-05	3.25E-01	1.80E-04
rs805303	rs12511169	0.346	0.365	2.05E-01	1.36E-04	1.84E-05	2.06E-01	3.07E-03
rs11776767	rs929280	0.38	0.038	3.54E-01	6.15E-04	2.86E-05	1.00E+00	3.79E-03
rs12328675	rs2406422	0.137	0.265	6.30E-01	5.06E-04	3.23E-05	3.76E-01	2.31E-03
rs4846914	rs974819	0.404	0.292	5.83E-02	7.01E-04	3.58E-05	3.92E-02	5.44E-04
rs11920090	rs890022	0.15	0.109	5.32E-01	7.38E-04	4.44E-05	2.18E-01	5.09E-01
rs693	rs7085495	0.461	0.004	8.61E-01	2.56E-04	5.05E-05	2.00E-01	1.25E-01
rs3846662	rs1457480	0.44	0.089	1.51E-01	1.54E-04	5.60E-05	5.90E-01	3.33E-02
rs2166706	rs17202030	0.406	0.444	6.41E-01	8.05E-04	5.96E-05	8.00E-01	1.56E-02
rs1746048	rs4864534	0.173	0.016	1.61E-04	3.04E-04	6.75E-05	1.42E-01	1.54E-01
rs7961581	rs11227513	0.307	0.099	3.72E-01	4.11E-04	8.07E-05	5.60E-01	7.66E-03
rs16942887	rs12619970	0.119	0.282	1.53E-01	3.59E-04	8.09E-05	--	--
rs3905000	rs7161989	0.145	0.262	4.22E-01	3.01E-04	8.10E-05	1.09E-01	6.94E-03
rs1333049	rs4686947	0.483	0.167	3.42E-07	2.83E-04	8.28E-05	1.33E-01	1.41E-05
rs7826222	rs4876804	0.167	0.266	6.58E-01	8.05E-04	9.24E-05	--	--
rs12936587	rs7940379	0.434	0.204	6.18E-01	9.33E-04	9.34E-05	8.46E-01	2.92E-03
rs1530440	rs12941859	0.187	0.205	9.44E-01	3.82E-04	9.38E-05	8.07E-01	1.02E-02
rs1531343	rs6000401	0.141	0.026	9.45E-01	2.28E-04	9.40E-05	8.09E-01	1.16E-01
rs340874	rs1573809	0.487	0.055	4.65E-01	6.01E-04	1.05E-04	2.58E-01	6.68E-04
rs10423928	rs299467	0.097	0.311	1.40E-03	7.61E-04	1.05E-04	1.40E-01	4.48E-03
rs11605924	rs12346989	0.468	0.048	8.14E-01	6.43E-04	1.08E-04	4.94E-01	4.33E-01
rs3741414	rs12641856	0.137	0.053	4.67E-01	6.08E-04	1.09E-04	3.38E-01	4.66E-02
rs28927680	rs4490836	0.074	0.475	1.28E-01	6.68E-04	1.22E-04	1.66E-01	1.96E-02
rs6548238	rs12595857	0.19	0.51	9.02E-02	9.26E-04	1.26E-04	4.48E-01	2.26E-04
rs492602	rs17069996	0.424	0.058	7.13E-01	1.25E-04	1.28E-04	--	--
rs1329650	rs4876804	0.259	0.266	4.80E-01	8.05E-04	1.36E-04	8.03E-02	4.16E-04
rs11084753	rs12497236	0.351	0.099	2.42E-01	7.49E-04	1.45E-04	1.92E-01	2.00E-02
rs231362	rs736288	0.47	0.048	5.33E-01	1.00E-03	1.54E-04	2.60E-01	7.98E-03
rs4373814	rs10050400	0.443	0.028	1.11E-01	9.64E-04	1.58E-04	3.04E-01	8.30E-04
rs599839	rs12286002	0.213	0.056	7.77E-02	6.29E-04	1.73E-04	3.87E-01	1.06E-01
rs1689800	rs9939575	0.381	0.081	6.18E-02	7.73E-04	1.73E-04	7.94E-02	2.10E-01
rs16948048	rs751984	0.368	0.071	7.60E-03	3.23E-04	1.90E-04	7.55E-01	1.61E-02
rs3825807	rs12320080	0.438	0.083	4.40E-01	4.43E-05	1.90E-04	6.00E-01	5.73E-02
rs2237892	rs749146	0.052	0.465	8.15E-01	7.05E-04	2.02E-04	6.77E-02	3.62E-03
rs2072183	rs3794986	0.131	0.418	5.83E-02	4.89E-04	2.14E-04	6.35E-01	1.21E-02
rs649129	rs4298013	0.231	0.448	2.95E-03	4.72E-04	2.17E-04	8.93E-01	1.73E-02
rs2814944	rs4241895	0.145	0.21	3.80E-03	9.59E-04	2.23E-04	9.92E-01	1.53E-02
rs2605100	rs2890593	0.272	0.423	7.69E-01	8.15E-04	2.25E-04	5.88E-01	8.45E-04
rs7129220	rs11723612	0.116	0.296	6.38E-01	9.70E-04	2.36E-04	6.47E-01	4.02E-02
rs11136341	rs12806315	0.358	0.025	2.97E-01	1.91E-04	2.39E-04	6.20E-01	2.66E-02
rs11014166	rs10483099	0.375	0.166	6.43E-01	5.75E-04	2.50E-04	4.86E-01	6.92E-03
rs6015450	rs3112998	0.103	0.412	8.68E-01	4.19E-04	2.57E-04	7.61E-01	2.80E-02
rs2191349	rs10003420	0.452	0.043	7.05E-01	1.60E-05	2.66E-04	9.59E-01	1.22E-01
rs12190287	rs12211268	0.368	0.463	4.44E-01	9.60E-04	2.69E-04	4.60E-01	4.10E-02
rs3846662	rs4947084	0.44	0.127	1.51E-01	7.04E-04	2.80E-04	2.63E-01	1.13E-02
rs7515577	rs4917465	0.189	0.247	7.16E-01	5.97E-04	2.82E-04	8.28E-01	3.71E-03
rs2605100	rs7634628	0.272	0.172	7.69E-01	4.70E-04	2.86E-04	7.14E-02	2.50E-04
rs6474412	rs7272983	0.177	0.114	8.16E-02	3.58E-04	2.96E-04	9.45E-01	2.51E-01
rs4939883	rs9533737	0.159	0.33	5.54E-01	5.37E-04	3.05E-04	9.73E-01	1.69E-02

[a] Minor allele frequency in MIGen controls

[b] Data were available for both SNPs in this pair, but the meta-analysis model returned an unreliable result due to extreme variance in for some of the interaction terms

[c] p-value for association with MI in the MIGen study (adjusted for age, sex and IBS principal components; additive genetic model)

Analysis 3a

SNP1	SNP2	MAF SNP1[a]	MAF SNP2[a]	p-value for MI, SNP1[c]	p-value for MI, SNP2[c]	MIGen interaction p-value	WTCCC interaction p-value	Meta-analysis interaction p-value
rs761174	rs167490	0.257	0.016	1.75E-05	5.92E-04	3.49E-06	5.90E-03	9.63E-03
rs7614572	rs4241895	0.335	0.21	5.92E-04	9.59E-04	3.19E-05	9.23E-01	1.29E-01
rs17081749	rs11138270	0.09	0.071	4.75E-04	5.59E-04	3.88E-05	7.60E-01	2.66E-02
rs16920992	rs6540043	0.009	0.502	5.75E-04	8.03E-04	4.11E-05	9.51E-01	8.75E-02
rs2906289	rs2871006	0.481	0.296	9.49E-04	8.93E-04	5.44E-05	7.86E-02	1.06E-04
rs2513403	rs11616460	0.27	0.375	6.22E-04	8.09E-04	6.51E-05	8.74E-01	8.73E-03
rs2034441	rs7932813	0.196	0.183	7.56E-04	9.37E-04	6.71E-05	9.28E-01	2.50E-02
rs5882	rs2434853	0.286	0.101	3.10E-04	6.64E-04	6.78E-05	4.13E-01	1.15E-03
rs12941859	rs12626156	0.205	0.008	3.82E-04	2.50E-04	6.91E-05	--	--
rs1034383	rs12341867	0.413	0.044	3.66E-04	3.19E-05	7.19E-05	8.51E-01	7.20E-03
rs4233508	rs550517	0.302	0.478	7.57E-04	9.45E-04	7.56E-05	3.63E-01	7.13E-04
rs2353579	rs742487	0.511	0.061	8.83E-04	3.14E-04	7.85E-05	1.53E-01	3.60E-04
rs6852986	rs17149981	0.09	0.032	9.10E-05	1.82E-05	8.90E-05	1.51E-01	3.80E-02
rs10510786	rs7809551	0.395	0.209	3.28E-04	6.33E-04	8.95E-05	4.38E-01	2.59E-02
rs4696618	rs4767329	0.219	0.473	2.27E-04	3.50E-04	9.31E-05	--	--
rs12674115	rs10492761	0.19	0.461	5.36E-04	7.59E-04	1.12E-04	7.43E-01	1.01E-02
rs969368	rs8087353	0.074	0.268	5.75E-04	5.69E-04	1.13E-04	6.85E-01	2.51E-02
rs17360414	rs1909218	0.056	0.153	6.44E-04	9.40E-04	1.14E-04	3.09E-01	4.02E-03
rs2324982	rs1870146	0.039	0.11	7.21E-04	5.14E-04	1.15E-04	9.72E-01	1.33E-01
rs3765857	rs477262	0.456	0.307	4.34E-05	9.88E-04	1.23E-04	--	--
rs10239003	rs7161989	0.372	0.262	5.33E-04	3.01E-04	1.26E-04	3.77E-01	4.11E-02
rs10510786	rs10050400	0.395	0.028	3.28E-04	9.64E-04	1.29E-04	3.19E-01	8.37E-04
rs8011392	rs3790076	0.2	0.439	5.83E-04	7.89E-04	1.35E-04	8.28E-01	1.82E-02
rs9990208	rs1570647	0.091	0.119	1.20E-04	9.04E-04	1.39E-04	2.28E-01	1.24E-01
rs17350838	rs7193186	0.221	0.076	3.68E-04	7.38E-04	1.47E-04	4.83E-01	3.92E-03
rs2295514	rs442965	0.12	0.209	8.45E-04	5.44E-04	1.48E-04	3.46E-01	3.75E-02
rs2930382	rs17089546	0.346	0.246	3.40E-04	5.17E-06	1.53E-04	8.92E-01	2.44E-02
rs606452	rs289742	0.146	0.148	8.25E-04	5.87E-05	1.76E-04	3.43E-01	3.83E-03
rs7830977	rs5882	0.254	0.286	5.36E-04	3.10E-04	1.77E-04	--	--
rs12529747	rs17735525	0.176	0.08	2.71E-04	8.33E-04	1.78E-04	3.01E-01	5.53E-02
rs12672541	rs12626156	0.408	0.008	9.54E-04	2.50E-04	1.82E-04	9.98E-01	--[b]
rs6852986	rs4767329	0.09	0.473	9.10E-05	3.50E-04	1.86E-04	6.00E-01	1.11E-01
rs12497236	rs12626156	0.099	0.008	7.49E-04	2.50E-04	1.89E-04	--	--
rs6578453	rs1345117	0.061	0.43	2.57E-05	2.86E-05	1.97E-04	3.69E-01	3.61E-03
rs1407837	rs17619273	0.229	0.031	5.86E-05	6.40E-04	2.00E-04	8.22E-01	2.50E-02
rs4233508	rs10239003	0.302	0.372	7.57E-04	5.33E-04	2.04E-04	2.93E-02	1.63E-01
rs12120351	rs9316444	0.011	0.275	7.73E-04	6.36E-04	2.08E-04	6.75E-01	4.79E-02
rs1486563	rs11656173	0.505	0.4	8.37E-04	6.88E-04	2.08E-04	5.24E-01	2.65E-02
rs7138263	rs11179868	0.23	0.101	6.90E-04	8.29E-04	2.09E-04	5.43E-01	7.99E-03
rs1839022	rs9577914	0.18	0.309	4.74E-04	9.80E-04	2.17E-04	--	--
rs234029	rs10811650	0.041	0.514	5.86E-04	7.72E-07	2.20E-04	3.39E-01	4.46E-03
rs7550312	rs974819	0.008	0.292	7.54E-04	7.01E-04	2.22E-04	--	--
rs12211268	rs7927116	0.463	0.007	9.60E-04	1.14E-04	2.33E-04	7.56E-01	2.18E-02
rs7518519	rs467634	0.291	0.137	8.63E-04	9.95E-04	2.43E-04	2.47E-01	1.55E-04
rs2182861	rs11660701	0.398	0.423	6.50E-04	5.23E-04	2.49E-04	9.35E-01	7.96E-03
rs4298013	rs12529747	0.448	0.176	4.72E-04	2.71E-04	2.72E-04	2.42E-02	1.93E-03
rs4696618	rs17470826	0.219	0.042	2.27E-04	9.00E-04	2.72E-04	--	--
rs925669	rs11656173	0.425	0.4	1.91E-04	6.88E-04	2.76E-04	1.27E-01	1.66E-01
rs12529747	rs1788823	0.176	0.365	2.71E-04	6.94E-04	2.77E-04	2.09E-01	6.90E-04
rs4241895	rs10827949	0.21	0.257	9.59E-04	4.07E-04	2.85E-04	6.54E-01	2.51E-02
rs12511169	rs289742	0.365	0.148	1.36E-04	5.87E-05	2.85E-04	4.69E-01	4.75E-02
rs17202030	rs16956631	0.444	0.043	8.05E-04	6.51E-04	2.86E-04	6.02E-01	3.01E-02
rs17167126	rs11212823	0.04	0.147	4.91E-05	7.97E-04	2.87E-04	5.83E-01	2.62E-02
rs232540	rs3020839	0.376	0.437	4.05E-04	7.95E-04	2.90E-04	7.84E-01	3.06E-02

[a] Minor allele frequency in MIGen controls

[b] Data were available for both SNPs in this pair, but the meta-analysis model returned an unreliable result due to extreme variance for some of the interaction terms

[c] p-value for association with MI in the MIGen study (adjusted for age, sex and IBS principal components; additive genetic model)

Analysis 3b

SNP1	SNP2	MAF SNP1[a]	MAF SNP2[a]	p-value for MI, SNP1[c]	p-value for MI, SNP2[c]	MIGen interaction p-value	WTCCC interaction p-value	Meta-analysis interaction p-value
rs194243	rs4589969	0.285	0.231	3.97E-03	7.75E-03	5.51E-08	9.44E-02	4.78E-05
rs2844477	rs12684383	0.402	0.151	7.17E-03	2.02E-03	1.31E-07	6.25E-01	2.13E-03
rs10496796	rs7660421	0.169	0.099	3.22E-03	9.55E-03	2.56E-07	--	--
rs6972638	rs7211960	0.198	0.235	1.91E-03	1.15E-03	3.38E-07	6.01E-02	7.01E-07
rs1414648	rs2203943	0.014	0.407	9.19E-03	4.20E-03	3.66E-07	3.50E-01	9.36E-03
rs6945902	rs7232613	0.221	0.005	6.26E-03	7.04E-03	3.98E-07	7.94E-02	3.83E-05
rs494620	rs12684383	0.493	0.151	1.00E-02	2.02E-03	4.57E-07	3.22E-01	3.39E-03
rs9458301	rs10237218	0.451	0.414	5.71E-03	8.30E-03	5.44E-07	--	--
rs2290853	rs6754251	0.011	0.264	8.06E-03	5.93E-03	5.78E-07	4.81E-01	1.20E-01
rs7603414	rs2995214	0.042	0.219	9.80E-03	5.77E-03	6.99E-07	--	--
rs2569248	rs11148656	0.436	0.152	3.08E-03	8.56E-03	8.10E-07	1.70E-01	6.47E-05
rs11209322	rs39387	0.43	0.435	9.49E-03	2.55E-03	8.63E-07	7.83E-01	7.64E-03
rs11540586	rs11760323	0.327	0.461	7.64E-03	6.07E-03	8.98E-07	8.86E-01	1.41E-02
rs7009235	rs12460041	0.427	0.062	5.82E-03	7.50E-03	9.18E-07	1.20E-01	2.54E-02
rs884799	rs12155347	0.123	0.024	5.30E-03	7.14E-03	1.06E-06	7.12E-01	1.77E-03
rs6796681	rs10520025	0.1	0.153	7.81E-03	3.40E-03	1.10E-06	5.22E-01	5.89E-03
rs767664	rs11914212	0.247	0.107	7.57E-03	4.42E-03	1.13E-06	4.84E-01	1.01E-03
rs12134558	rs1001415	0.022	0.094	8.88E-03	3.09E-03	1.17E-06	1.80E-01	9.85E-04
rs6599272	rs9341904	0.117	0.485	5.69E-03	4.20E-03	1.20E-06	9.24E-01	2.82E-03
rs7206390	rs1704497	0.163	0.422	4.18E-03	3.24E-03	1.24E-06	7.61E-01	7.69E-04
rs12636662	rs9949270	0.365	0.022	7.42E-03	2.09E-03	1.27E-06	3.46E-01	6.35E-03
rs11675475	rs4946000	0.476	0.013	4.63E-03	1.21E-03	1.28E-06	7.19E-01	4.84E-02
rs756465	rs11595511	0.298	0.015	8.68E-03	4.86E-03	1.45E-06	4.18E-01	1.27E-03
rs11579007	rs1383389	0.403	0.274	7.54E-03	6.47E-03	1.59E-06	4.19E-02	3.68E-05
rs13151220	rs2859369	0.096	0.091	1.00E-02	6.88E-03	1.62E-06	2.46E-01	1.15E-01
rs11660396	rs4945876	0.263	0.082	4.59E-03	5.29E-04	1.64E-06	1.32E-01	2.96E-03
rs4858670	rs17020483	0.334	0.073	3.20E-03	5.84E-03	1.71E-06	2.77E-01	1.78E-03
rs13257940	rs6867011	0.048	0.202	8.35E-03	6.46E-04	1.74E-06	7.29E-01	1.95E-03
rs10811032	rs10796850	0.091	0.33	1.64E-03	8.21E-03	1.75E-06	--	--
rs4619848	rs12249208	0.477	0.028	7.73E-03	3.25E-03	1.76E-06	9.69E-02	4.19E-05
rs17045713	rs12457257	0.126	0.324	8.59E-03	8.29E-03	1.77E-06	7.26E-01	5.56E-03
rs12052288	rs4696822	0.122	0.244	8.49E-03	6.91E-03	1.79E-06	6.39E-01	7.18E-03
rs7558386	rs212046	0.379	0.171	1.00E-02	9.25E-03	1.87E-06	1.42E-01	1.65E-02
rs12995732	rs16881257	0.074	0.08	2.70E-03	9.73E-03	1.94E-06	1.35E-01	4.06E-03
rs12732279	rs2007324	0.397	0.439	7.34E-03	5.43E-03	1.96E-06	1.06E-01	1.03E-02
rs2258180	rs4667972	0.485	0.244	5.05E-03	9.74E-04	2.00E-06	2.27E-01	1.19E-02
rs1479027	rs3115512	0.115	0.142	6.49E-03	9.90E-03	2.08E-06	6.93E-01	2.33E-03
rs41561	rs11703137	0.027	0.153	6.30E-03	8.22E-04	2.12E-06	3.50E-01	1.55E-01
rs12244105	rs12277517	0.114	0.027	5.04E-03	1.28E-03	2.23E-06	6.30E-02	1.34E-01
rs243069	rs4734582	0.36	0.144	7.54E-03	6.79E-03	2.25E-06	4.42E-01	3.38E-02
rs6557475	rs4976349	0.107	0.046	9.01E-03	3.70E-04	2.26E-06	8.00E-01	5.45E-02
rs643064	rs4872179	0.072	0.452	3.11E-03	4.86E-04	2.33E-06	3.30E-01	3.42E-03
rs4658345	rs4459626	0.019	0.215	8.41E-03	3.97E-03	2.35E-06	3.30E-01	6.82E-03
rs138400	rs5747997	0.19	0.485	8.87E-03	5.00E-04	2.37E-06	--	--
rs227723	rs2058318	0.284	0.382	7.28E-03	5.59E-04	2.56E-06	5.03E-01	7.64E-02
rs7350481	rs7276176	0.052	0.506	3.22E-03	8.97E-03	2.64E-06	5.55E-01	1.19E-02
rs17151028	rs10898329	0.32	0.361	4.21E-03	1.90E-03	2.79E-06	4.04E-01	3.52E-04
rs3971872	rs3788437	0.046	0.167	8.38E-03	3.14E-03	2.91E-06	4.51E-01	1.67E-02
rs867434	rs2302984	0.191	0.103	6.86E-03	7.23E-03	3.12E-06	1.15E-02	1.26E-03
rs13008578	rs16951125	0.164	0.09	4.00E-03	3.19E-03	3.15E-06	7.11E-02	1.33E-02
rs1864933	rs11641045	0.493	0.104	6.52E-03	2.26E-03	3.23E-06	6.56E-01	1.32E-03
rs7176821	rs1865093	0.086	0.093	6.79E-03	4.28E-03	3.37E-06	8.51E-01	8.46E-04
rs1421596	rs17677649	0.15	0.165	4.22E-03	9.65E-03	3.47E-06	4.44E-01	5.15E-04
rs3733471	rs6534832	0.218	0.352	7.50E-03	7.42E-03	3.48E-06	9.89E-01	1.51E-02
rs7560239	rs6446762	0.421	0.019	4.36E-03	7.68E-03	3.49E-06	5.09E-01	2.95E-02

[a] Minor allele frequency in MIGen controls

[b] Data were available for both SNPs in this pair, but the meta-analysis model returned an unreliable result due to extreme variance in for some of the interaction terms

[c] p-value for association with MI in the MIGen study (adjusted for age, sex and IBS principal components; additive genetic model)

Table 3. Power computation.

Effect sizes ($\beta^{0.8}$) for pairs of SNPs with MAFs between 0.02 and 0.5, under a additive × additive interaction model (results for other models not shown). '--' denotes instances where the effect size could not be calculated for any of the SNP pairs sampled because of the low frequency of the double rare homozygote. See S3.6 for details of computation and S.F4 for a graphical representation of these results, and also for dominant × dominant and recessive × recessive interaction models.

ANALYSIS 1. Additive × additive model

MAF	(0.02,0.04]	(0.04,0.06]	(0.06,0.08]	(0.08,0.1]	(0.1,0.12]	(0.12,0.14]	(0.14,0.16]	(0.16,0.18]	(0.18,0.2]	(0.2,0.22]	(0.22,0.24]	(0.24,0.26]	(0.26,0.28]	(0.28,0.3]	(0.3,0.32]	(0.32,0.34]	(0.34,0.36]	(0.36,0.38]	(0.38,0.4]	(0.4,0.42]	(0.42,0.44]	(0.44,0.46]	(0.46,0.48]	(0.48,0.5]
**(0.02,0.04]**	--	--	--	--	--	--	--	--	--	--	--	--	--	--	--	--	--	--	--	--	--	--	--	--
**(0.04,0.06]**	--	--	--	--	--	--	--	--	--	--	--	--	--	--	--	2.12	--	--	--	--	--	--	2.03	--
**(0.06,0.08]**	--	--	--	--	--	--	--	--	--	--	--	--	--	--	--	--	1.89	--	1.90	1.89	--	1.89	1.87	1.84
**(0.08,0.1]**	--	--	--	--	2.57	--	--	--	--	--	--	--	--	--	1.86	--	--	--	--	--	1.79	1.79	1.80	1.77
**(0.1,0.12]**	--	--	--	2.57	--	--	--	--	--	--	--	--	--	--	--	--	1.74	--	1.72	1.71	1.72	--	1.72	1.70
**(0.12,0.14]**	--	--	--	--	--	--	--	--	--	--	1.76	--	1.72	1.70	1.66	1.66	1.65	1.64	1.64	1.62	1.61	1.62	1.61	1.62
**(0.14,0.16]**	--	--	--	--	--	--	--	1.83	--	1.76	--	1.68	1.68	1.65	1.63	1.62	1.61	1.61	1.60	1.59	1.59	1.58	1.59	1.57
**(0.16,0.18]**	--	--	--	--	--	--	1.83	--	--	--	1.67	1.64	1.63	1.60	1.59	1.58	1.58	1.56	1.56	1.55	1.55	1.53	1.53	1.54
**(0.18,0.2]**	--	--	--	--	--	--	--	--	1.70	1.66	1.65	1.61	1.60	1.58	1.56	1.55	1.55	1.54	1.53	1.53	1.52	1.52	1.52	1.52
**(0.2,0.22]**	--	--	--	--	--	--	1.76	--	1.66	1.63	1.61	1.58	1.58	1.56	1.54	1.53	1.52	1.51	1.52	1.50	1.50	1.50	1.50	1.48
**(0.22,0.24]**	--	--	--	--	--	1.76	--	1.67	1.65	1.61	1.59	1.56	1.55	1.55	1.52	1.51	1.51	1.50	1.49	1.48	1.47	1.48	1.48	1.47
**(0.24,0.26]**	--	--	--	--	--	--	1.68	1.64	1.61	1.58	1.56	1.54	1.52	1.52	1.50	1.48	1.48	1.47	1.46	1.46	1.46	1.45	1.46	1.46
**(0.26,0.28]**	--	--	--	--	--	1.72	1.68	1.63	1.60	1.58	1.55	1.52	1.51	1.51	1.48	1.47	1.47	1.46	1.46	1.45	1.45	1.44	1.45	1.44
**(0.28,0.3]**	--	--	--	--	--	1.70	1.65	1.60	1.58	1.56	1.55	1.52	1.51	1.49	1.47	1.47	1.46	1.46	1.45	1.45	1.44	1.44	1.43	1.44
**(0.3,0.32]**	--	--	--	1.86	--	1.66	1.63	1.59	1.56	1.54	1.52	1.50	1.48	1.47	1.46	1.45	1.45	1.43	1.43	1.43	1.43	1.42	1.42	1.42
**(0.32,0.34]**	--	2.12	--	--	--	1.66	1.62	1.58	1.55	1.53	1.51	1.48	1.47	1.47	1.45	1.44	1.44	1.43	1.42	1.42	1.41	1.42	1.42	1.41
**(0.34,0.36]**	--	--	1.89	--	1.74	1.65	1.61	1.58	1.55	1.52	1.51	1.48	1.47	1.46	1.45	1.44	1.42	1.42	1.42	1.41	1.41	1.41	1.40	1.40
**(0.36,0.38]**	--	--	--	--	--	1.64	1.61	1.56	1.54	1.51	1.50	1.47	1.46	1.46	1.43	1.43	1.42	1.42	1.41	1.41	1.40	1.40	1.40	1.40
**(0.38,0.4]**	--	--	1.90	--	1.72	1.64	1.60	1.56	1.53	1.52	1.49	1.46	1.46	1.45	1.43	1.42	1.42	1.41	1.41	1.41	1.40	1.40	1.39	1.40
**(0.4,0.42]**	--	--	1.89	--	1.71	1.62	1.59	1.55	1.53	1.50	1.48	1.46	1.45	1.45	1.43	1.42	1.41	1.41	1.41	1.40	1.39	1.39	1.39	1.39
**(0.42,0.44]**	--	--	--	1.79	1.72	1.61	1.59	1.55	1.52	1.50	1.47	1.46	1.45	1.44	1.43	1.41	1.41	1.40	1.40	1.39	1.39	1.39	1.38	1.38
**(0.44,0.46]**	--	--	1.89	1.79	--	1.62	1.58	1.53	1.52	1.50	1.48	1.45	1.44	1.44	1.42	1.42	1.41	1.40	1.40	1.39	1.39	1.39	1.39	1.39
**(0.46,0.48]**	--	2.03	1.87	1.80	1.72	1.61	1.59	1.53	1.52	1.50	1.48	1.46	1.45	1.43	1.42	1.42	1.40	1.40	1.39	1.39	1.38	1.39	1.39	1.39
**(0.48,0.5]**	--	--	1.84	1.77	1.70	1.62	1.57	1.54	1.52	1.48	1.47	1.46	1.44	1.44	1.42	1.41	1.40	1.40	1.40	1.39	1.38	1.39	1.39	1.38

ANALYSIS 2. Additive × additive model

MAF	(0.02,0.04]	(0.04,0.06]	(0.06,0.08]	(0.08,0.1]	(0.1,0.12]	(0.12,0.14]	(0.14,0.16]	(0.16,0.18]	(0.18,0.2]	(0.2,0.22]	(0.22,0.24]	(0.24,0.26]	(0.26,0.28]	(0.28,0.3]	(0.3,0.32]	(0.32,0.34]	(0.34,0.36]	(0.36,0.38]	(0.38,0.4]	(0.4,0.42]	(0.42,0.44]	(0.44,0.46]	(0.46,0.48]	(0.48,0.5]
**(0.02,0.04]**	--	--	--	--	--	--	--	--	--	--	--	--	--	--	--	--	--	--	--	--	--	--	--	--
**(0.04,0.06]**	--	--	--	--	--	--	--	--	--	--	--	--	--	--	--	--	--	--	--	--	--	--	--	--
**(0.06,0.08]**	--	--	--	--	--	--	--	--	--	--	--	--	--	--	--	--	--	--	--	--	--	--	--	--
**(0.08,0.1]**	--	--	--	--	--	--	--	--	--	--	--	--	--	--	--	--	--	--	--	--	--	--	--	1.91
**(0.1,0.12]**	--	--	--	--	--	--	--	--	--	--	--	--	--	--	--	--	--	--	--	--	1.83	1.84	1.83	--
**(0.12,0.14]**	--	--	--	--	--	--	--	--	--	--	--	--	--	--	--	--	--	--	1.75	1.74	--	1.74	1.74	1.73
**(0.14,0.16]**	--	--	--	--	--	--	--	--	--	--	--	--	--	1.75	1.73	--	1.70	1.69	1.67	1.68	1.68	1.68	1.61	1.66
**(0.16,0.18]**	--	--	--	--	--	--	--	--	--	1.85	--	1.72	1.75	1.72	1.69	1.68	1.67	1.65	1.62	1.64	1.62	1.62	1.61	1.60
**(0.18,0.2]**	--	--	--	--	--	--	--	--	--	1.76	--	1.71	1.67	1.65	1.64	1.63	1.61	1.60	1.60	1.58	1.59	1.58	1.57	1.57
**(0.2,0.22]**	--	--	--	--	--	--	--	1.85	1.76	--	1.70	1.68	1.66	1.63	1.62	1.61	1.59	1.59	1.57	1.57	1.56	1.56	1.56	1.54
**(0.22,0.24]**	--	--	--	--	--	--	--	--	--	1.70	--	1.65	1.63	1.60	1.60	1.56	1.56	1.55	1.55	1.54	1.53	1.53	1.52	1.51
**(0.24,0.26]**	--	--	--	--	--	--	--	1.72	1.71	1.68	1.65	1.63	1.61	1.57	1.56	1.55	1.54	1.52	1.53	1.51	1.51	1.50	1.50	1.51
**(0.26,0.28]**	--	--	--	--	--	--	--	1.75	1.67	1.66	1.63	1.61	1.58	1.55	1.54	1.52	1.51	1.51	1.50	1.49	1.50	1.48	1.49	1.48
**(0.28,0.3]**	--	--	--	--	--	--	1.75	1.72	1.65	1.63	1.60	1.57	1.55	1.55	1.53	1.51	1.51	1.49	1.49	1.49	1.48	1.48	1.48	1.47
**(0.3,0.32]**	--	--	--	--	--	--	1.73	1.69	1.64	1.62	1.60	1.56	1.54	1.53	1.52	1.50	1.49	1.49	1.47	1.47	1.47	1.45	1.46	1.46
**(0.32,0.34]**	--	--	--	--	--	--	--	1.68	1.63	1.61	1.56	1.55	1.52	1.51	1.50	1.48	1.48	1.47	1.47	1.46	1.46	1.45	1.44	1.45
**(0.34,0.36]**	--	--	--	--	--	--	1.70	1.67	1.61	1.59	1.56	1.54	1.51	1.51	1.49	1.48	1.46	1.44	1.46	1.44	1.45	1.43	1.44	1.44
**(0.36,0.38]**	--	--	--	--	--	--	1.69	1.65	1.60	1.59	1.55	1.52	1.51	1.49	1.49	1.47	1.44	1.46	1.45	1.45	1.44	1.44	1.44	1.43
**(0.38,0.4]**	--	--	--	--	--	1.75	1.67	1.62	1.60	1.57	1.55	1.53	1.50	1.49	1.47	1.47	1.46	1.45	1.44	1.43	1.43	1.42	1.42	1.42
**(0.4,0.42]**	--	--	--	--	--	1.74	1.68	1.64	1.58	1.57	1.54	1.51	1.49	1.49	1.47	1.46	1.44	1.45	1.43	1.43	1.43	1.40	1.42	1.42
**(0.42,0.44]**	--	--	--	--	1.83	--	1.68	1.62	1.59	1.56	1.53	1.51	1.50	1.48	1.47	1.46	1.45	1.44	1.43	1.43	1.42	1.42	1.42	1.41
**(0.44,0.46]**	--	--	--	--	1.84	1.74	1.68	1.62	1.58	1.56	1.53	1.50	1.48	1.48	1.45	1.45	1.43	1.44	1.42	1.40	1.42	1.40	1.41	1.41
**(0.46,0.48]**	--	--	--	--	1.83	1.74	1.61	1.61	1.57	1.56	1.52	1.50	1.49	1.48	1.46	1.44	1.44	1.44	1.42	1.42	1.42	1.41	1.41	1.41
**(0.48,0.5]**	--	--	--	1.91	--	1.73	1.66	1.60	1.57	1.54	1.51	1.51	1.48	1.47	1.46	1.45	1.44	1.43	1.42	1.42	1.41	1.41	1.41	1.41

ANALYSIS 3a (marginal SNPs $p<10^{-3}$). Additive × additive model

MAF	(0.02,0.04]	(0.04,0.06]	(0.06,0.08]	(0.08,0.1]	(0.1,0.12]	(0.12,0.14]	(0.14,0.16]	(0.16,0.18]	(0.18,0.2]	(0.2,0.22]	(0.22,0.24]	(0.24,0.26]	(0.26,0.28]	(0.28,0.3]	(0.3,0.32]	(0.32,0.34]	(0.34,0.36]	(0.36,0.38]	(0.38,0.4]	(0.4,0.42]	(0.42,0.44]	(0.44,0.46]	(0.46,0.48]	(0.48,0.5]
**(0.02,0.04]**	--	--	--	--	--	--	--	--	--	5.45	--	4.51	--	--	--	--	--	--	--	--	--	--	--	--
**(0.04,0.06]**	--	--	--	--	--	--	--	--	--	--	--	--	--	--	--	--	--	--	--	--	--	--	--	--
**(0.06,0.08]**	--	--	--	--	--	--	--	--	--	--	--	--	--	--	--	--	--	--	--	--	--	--	--	--
**(0.08,0.1]**	--	--	--	--	--	--	--	--	--	--	--	--	--	--	--	--	--	--	--	1.95	--	--	--	--
**(0.1,0.12]**	--	--	--	--	--	--	--	--	--	--	--	--	--	--	--	--	1.89	--	--	1.84	1.80	1.83	1.80	1.84
**(0.12,0.14]**	--	--	--	--	--	--	--	--	--	--	--	--	1.86	--	--	1.81	1.70	1.75	1.76	--	1.73	1.72	1.72	1.70
**(0.14,0.16]**	--	--	--	--	--	--	--	--	--	--	--	--	--	1.77	1.75	--	--	1.69	1.69	1.68	1.67	1.66	1.67	1.68
**(0.16,0.18]**	--	--	--	--	--	--	--	--	--	1.83	--	1.76	--	1.72	--	1.65	1.63	1.64	1.64	1.64	1.63	1.59	1.61	1.60
**(0.18,0.2]**	--	--	--	--	--	--	--	--	1.82	1.77	1.76	1.69	1.69	1.67	1.64	1.62	1.62	1.60	1.59	1.58	1.59	1.58	1.57	1.57
**(0.2,0.22]**	5.45	--	--	--	--	--	--	1.83	1.77	1.73	--	1.67	1.66	1.65	--	1.61	1.58	1.57	1.57	1.57	1.56	1.53	1.55	1.55
**(0.22,0.24]**	--	--	--	--	--	--	--	--	1.76	--	1.67	1.64	1.62	1.62	1.60	1.57	1.56	1.54	1.55	1.54	1.53	1.53	1.53	1.52
**(0.24,0.26]**	4.51	--	--	--	--	--	--	1.76	1.69	1.67	1.64	1.60	1.58	1.59	1.57	1.55	1.55	1.53	1.52	1.52	1.50	1.51	1.51	1.50
**(0.26,0.28]**	--	--	--	--	--	1.86	--	--	1.69	1.66	1.62	1.58	1.57	1.55	1.54	1.52	1.52	1.51	1.50	1.50	1.49	1.49	1.48	1.48
**(0.28,0.3]**	--	--	--	--	--	--	1.77	1.72	1.67	1.65	1.62	1.59	1.55	1.54	1.52	1.52	1.47	1.55	1.48	1.48	1.48	1.48	1.47	1.46
**(0.3,0.32]**	--	--	--	--	--	--	1.75	--	1.64	--	1.60	1.57	1.54	1.52	1.52	1.51	1.49	1.49	1.47	1.47	1.46	1.47	1.46	1.45
**(0.32,0.34]**	--	--	--	--	--	1.81	--	1.65	1.62	1.61	1.57	1.55	1.52	1.52	1.51	1.47	1.49	1.48	1.47	1.46	1.45	1.45	1.45	1.44
**(0.34,0.36]**	--	--	--	--	1.89	1.70	--	1.63	1.62	1.58	1.56	1.55	1.52	1.47	1.49	1.49	1.46	1.47	1.46	1.45	1.45	1.44	1.43	1.43
**(0.36,0.38]**	--	--	--	--	--	1.75	1.69	1.64	1.60	1.57	1.54	1.53	1.51	1.55	1.49	1.48	1.47	1.45	1.45	1.45	1.44	1.41	1.43	1.43
**(0.38,0.4]**	--	--	--	--	--	1.76	1.69	1.64	1.59	1.57	1.55	1.52	1.50	1.48	1.47	1.47	1.46	1.45	1.44	1.43	1.43	1.43	1.42	1.46
**(0.4,0.42]**	--	--	--	1.95	1.84	--	1.68	1.64	1.58	1.57	1.54	1.52	1.50	1.48	1.47	1.46	1.45	1.45	1.43	1.43	1.43	1.43	1.42	1.42
**(0.42,0.44]**	--	--	--	--	1.80	1.73	1.67	1.63	1.59	1.56	1.53	1.50	1.49	1.48	1.46	1.45	1.45	1.44	1.43	1.43	1.42	1.38	1.41	1.41
**(0.44,0.46]**	--	--	--	--	1.83	1.72	1.66	1.59	1.58	1.53	1.53	1.51	1.49	1.48	1.47	1.45	1.44	1.41	1.43	1.43	1.38	1.41	1.41	1.40
**(0.46,0.48]**	--	--	--	--	1.80	1.72	1.67	1.61	1.57	1.55	1.53	1.51	1.48	1.47	1.46	1.45	1.43	1.43	1.42	1.42	1.41	1.41	1.41	1.40
**(0.48,0.5]**	--	--	--	--	1.84	1.70	1.68	1.60	1.57	1.55	1.52	1.50	1.48	1.46	1.45	1.44	1.43	1.43	1.46	1.42	1.41	1.40	1.40	1.41

ANALYSIS 3b (marginal SNPs $p<10^{-2}$). Additive × additive model

MAF	(0.02,0.04]	(0.04,0.06]	(0.06,0.08]	(0.08,0.1]	(0.1,0.12]	(0.12,0.14]	(0.14,0.16]	(0.16,0.18]	(0.18,0.2]	(0.2,0.22]	(0.22,0.24]	(0.24,0.26]	(0.26,0.28]	(0.28,0.3]	(0.3,0.32]	(0.32,0.34]	(0.34,0.36]	(0.36,0.38]	(0.38,0.4]	(0.4,0.42]	(0.42,0.44]	(0.44,0.46]	(0.46,0.48]	(0.48,0.5]
**(0.02,0.04]**	--	--	--	--	--	--	--	--	--	--	--	--	--	--	--	--	--	--	--	3.93	--	--	--	--
**(0.04,0.06]**	--	--	--	--	--	--	--	--	--	--	6.59	--	--	--	--	--	--	--	--	--	--	--	--	--
**(0.06,0.08]**	--	--	--	--	--	--	--	--	--	--	--	--	--	--	--	--	--	--	--	--	--	--	--	--
**(0.08,0.1]**	--	--	--	2.74	--	--	--	--	--	--	--	--	--	--	--	--	--	--	--	--	--	2.05	--	--
**(0.1,0.12]**	--	--	--	--	--	--	--	--	--	--	--	--	--	2.03	--	--	--	2.03	1.96	1.99	1.97	1.99	1.97	1.96
**(0.12,0.14]**	--	--	--	--	--	--	--	--	--	--	--	--	--	--	1.91	1.85	1.90	1.92	1.89	1.87	--	1.82	1.86	1.84
**(0.14,0.16]**	--	--	--	--	--	--	--	--	--	--	--	--	--	--	1.83	1.85	1.84	1.74	1.78	1.80	1.79	1.77	1.73	1.81
**(0.16,0.18]**	--	--	--	--	--	--	--	--	--	--	--	--	1.86	1.84	--	1.77	1.69	1.75	1.74	1.73	1.74	1.71	1.71	1.72
**(0.18,0.2]**	--	--	--	--	--	--	--	--	--	1.88	--	--	1.81	1.79	1.75	1.69	1.71	1.69	1.71	1.68	1.67	1.64	1.66	1.65
**(0.2,0.22]**	--	--	--	--	--	--	--	--	1.88	--	1.84	1.78	1.78	1.74	1.69	1.69	1.67	1.66	1.66	1.66	1.65	1.64	1.64	1.63
**(0.22,0.24]**	--	6.59	--	--	--	--	--	--	--	1.84	1.78	1.76	1.71	1.69	1.68	1.63	1.65	1.63	1.62	1.63	1.62	1.60	1.61	1.61
**(0.24,0.26]**	--	--	--	--	--	--	--	--	--	1.78	1.76	1.73	1.69	1.66	1.64	1.59	1.64	1.61	1.60	1.59	1.58	1.59	1.58	1.57
**(0.26,0.28]**	--	--	--	--	--	--	--	1.86	1.81	1.78	1.71	1.69	1.66	1.65	1.64	1.59	1.57	1.58	1.58	1.58	1.57	1.55	1.55	1.56
**(0.28,0.3]**	--	--	--	--	2.03	--	--	1.84	1.79	1.74	1.69	1.66	1.65	1.62	1.62	1.60	1.59	1.57	1.57	1.56	1.56	1.54	1.54	1.54
**(0.3,0.32]**	--	--	--	--	--	1.91	1.83	--	1.75	1.69	1.68	1.64	1.64	1.62	1.60	1.58	1.58	1.55	1.54	1.56	1.54	1.51	1.53	1.52
**(0.32,0.34]**	--	--	--	--	--	1.85	1.85	1.77	1.69	1.69	1.63	1.59	1.59	1.60	1.58	1.54	1.52	1.55	1.53	1.51	1.51	1.50	1.52	1.49
**(0.34,0.36]**	--	--	--	--	--	1.90	1.84	1.69	1.71	1.67	1.65	1.64	1.57	1.59	1.58	1.52	1.53	1.53	1.52	1.52	1.49	1.48	1.48	1.49
**(0.36,0.38]**	--	--	--	--	2.03	1.92	1.74	1.75	1.69	1.66	1.63	1.61	1.58	1.57	1.55	1.55	1.53	1.52	1.51	1.51	1.50	1.47	1.50	1.50
**(0.38,0.4]**	--	--	--	--	1.96	1.89	1.78	1.74	1.71	1.66	1.62	1.60	1.58	1.57	1.54	1.53	1.52	1.51	1.50	1.50	1.50	1.48	1.49	1.49
**(0.4,0.42]**	3.93	--	--	--	1.99	1.87	1.80	1.73	1.68	1.66	1.63	1.59	1.58	1.56	1.56	1.51	1.52	1.51	1.50	1.50	1.49	1.49	1.48	1.48
**(0.42,0.44]**	--	--	--	--	1.97	--	1.79	1.74	1.67	1.65	1.62	1.58	1.57	1.56	1.54	1.51	1.49	1.50	1.50	1.49	1.48	1.47	1.48	1.47
**(0.44,0.46]**	--	--	--	2.05	1.99	1.82	1.77	1.71	1.64	1.64	1.60	1.59	1.55	1.54	1.51	1.50	1.48	1.47	1.48	1.49	1.47	1.47	1.47	1.47
**(0.46,0.48]**	--	--	--	--	1.97	1.86	1.73	1.71	1.66	1.64	1.61	1.58	1.55	1.54	1.53	1.52	1.48	1.50	1.49	1.48	1.48	1.47	1.46	1.47
**(0.48,0.5]**	--	--	--	--	1.96	1.84	1.81	1.72	1.65	1.63	1.61	1.57	1.56	1.54	1.52	1.49	1.49	1.50	1.49	1.48	1.47	1.47	1.47	1.46

Figures

Figure 1. Source literature and process for selection of cardiovascular risk factor SNPs. Details of references supporting the inclusion of the selected SNPs is provided in S.T1

	**NHGRI GWAS Catalogue** [a]	**Additional Studies** [b]
**Risk Factor Category**	*reported phenotypes*	*references*
Total cholesterol (*TC*)	Cholesterol; Cholesterol, total; Biochemical measures	Teslovich *et al.* **Biological, clinical and population relevance of 95 loci for blood lipids.** *Nature* (2010).
LDL cholesterol (*LDL*)	LDL cholesterol; Biochemical measures; LDL particle size	
HDL cholesterol (*HDL*)	HDL cholesterol; Biochemical measures ; HDL particle size	
Triglycerides (*TG*)	Triglycerides; Quantitative traits; Biochemical measures; Other metabolic traits; Hematological and biochemical traits	
Smoking (*SMK*)	Nicotine dependence; Smoking behavior; Smoking cessation	The Tobacco And Genetics Consortium. **Genome-wide meta-analyses identify multiple loci associated with smoking behavior.** *Nat Genet* (2010)[2].
Blood Pressure (*BP*)	Blood pressure; Diastolic blood pressure; Hypertension; Hypertension (young onset); Systolic blood pressure; Biomedical quantitative traits	Wain *et al.* **Genome-wide association study identifies six new loci influencing pulse pressure and mean arterial pressure.** *Nat Genet* (2011).<br>Ehret *et al.* **Genetic variants in novel pathways influence blood pressure and cardiovascular disease risk.** *Nature* (2011).
Carbohydrate Metabolism (*CH;* incl. Type 2 Diabetes)	Diabetes (incident); Type 2 diabetes; Type 2 diabetes and 6 quantitative traits; Type 2 diabetes and other traits ; Diabetes related insulin traits; Fasting plasma glucose; Insulin response; Insulin traits; Other metabolic traits; Fasting glucose-related traits; Fasting insulin-related traits; Two-hour glucose challenge	Dupuis *et al.* **New genetic loci implicated in fasting glucose homeostasis and their impact on type 2 diabetes risk.** *Nat Genet* (2010).<br>Voight *et al.* **Twelve type 2 diabetes susceptibility loci identified through large-scale association analysis.** *Nat Genet* (2010).
Obesity or Body Mass Index (*OB*)	Adiposity; Body mass index; Body mass index and fat mass; Obesity; Obesity-related traits; Obesity (early onset extreme); Obesity (extreme); Obesity and osteoporosis; Waist circumference; Waist circumference and related phenotypes; Waist circumference traits; Weight; Anthropometric traits; Biomedical quantitative traits; Body mass (lean)	
Plasma LPa levels (*LP(a)*)	Plasma Lp (a) levels	Chasman *et al.* **Forty-three loci associated with plasma lipoprotein size, concentration, and cholesterol content in genome-wide analysis.** *PLoS Genet* (2009).
Concentration of Small LDL Particles (*smallLDL*)		
Myocardial infarction or Coronary Artery Disease (*MI/CAD*)	Coronary artery calcification; Coronary artery disease; Coronary disease; Endothelial function traits; Major CVD; Myocardial infarction; Myocardial infarction (early onset); Arterial stiffness	Schunkert *et al.* **Large-scale association analysis identifies 13 new susceptibility loci for coronary artery disease.** *Nat Genet* (2011).

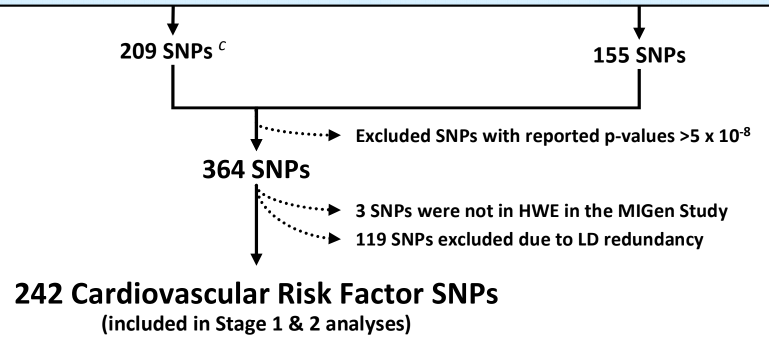

a. National Human Genome Research Institute Catalogue of Published Genome-Wide Association Studies[4], queried June 30th 2010.
b. Data from some relevant additional studies that were not included in the NHGRI catalogue on the date of our search were subsequently added to the list of CVRF SNPs. This is not an exhaustive list of all additional potentially relevant studies that have been published to date.
c. Querying the NHGRI catalogue (June 30th 2010) using the search terms shown in the 'Reported Phenotypes' column above returns more than 209 SNPs. This is because some search terms are of a general nature (e.g. biochemical measures, quantitative traits), and some of the results they return relate to specific sub-phenotypes that were not relevant for our analysis. We removed SNPs associated with these non-relevant phenotypes (unless they were also associated with phenotypes of interest), resulting in a list of 209 unique SNPs related to phenotypes of interest.

Figure 2. Graphical representation of interaction pairs tested in each Analysis.

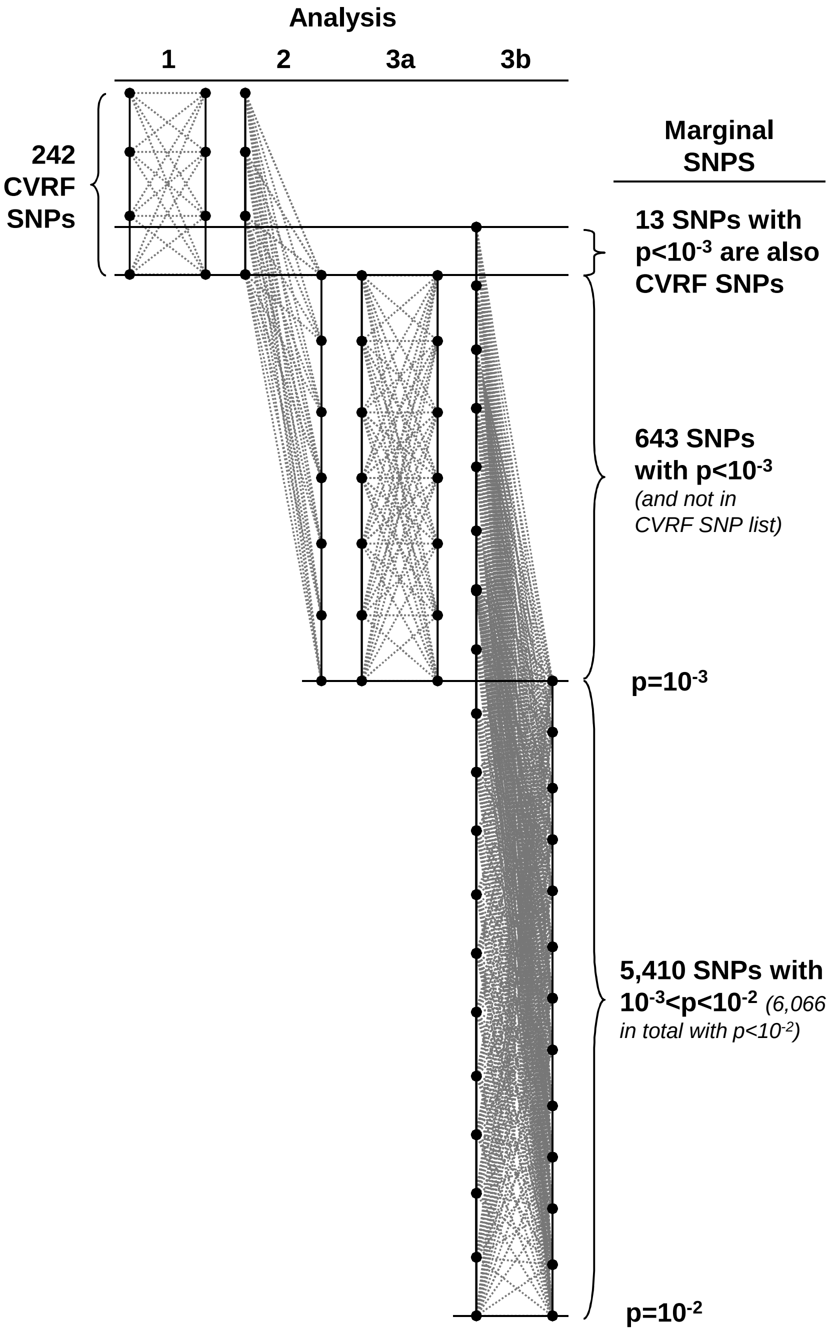

Sets of SNPs included in each Analysis are represented on the vertical axis (not to scale) and indicated by braces ('{' & '}'; CVRF SNP and marginal SNPs in the left and right columns, respectively). Individual pair-wise tests are represented schematically as dotted grey lines connecting the elements (black dots) of two lists of SNPs (represented by vertical lines). *Analysis 1*: 29,161 pair-wise tests among 242 CVRF SNPs; *Analysis 2*: 155,606 pair-wise tests between the 242 CVRF SNPs and the 643 SNPs that had marginal p-value$<10^{-3}$ for association with MI in MIGen and that were not included in Analysis 1; *Analysis 3a*: 206,403 pair-wise tests among the 643 marginal SNPs from Analysis 2; *Analysis 3b*: 18,180,305 pair-wise tests among 6,066 SNPs with marginal $p<10^{-2}$ in MIGen after excluding tests from previous Analyses.

Figure 3. Distribution of observed results with respect to their empirical expected null distribution, and computation of significance threshold to account for non-independence between tests (Analyses 2, 3a and 3b shown in rows 1-3 respectively; see Figure 2 in the main manuscript for results of the Analysis 1 analysis).

QQ-plots (left column). Quantile-quantile plots showing rank-ordered observed results (black points; *y-axis*) against expected results (*x-axis*) estimated from a large number of permutations of the analysis under the null hypothesis (randomized MI status). See S3.5 for computation methods. The shaded gray area corresponds to the 95% confidence interval of the permuted expected results. Note that, while our estimates of the expected results for Analyses 2-3 should be robust since they correspond to the medians for each rank, the boundaries of the 95% CI are less stable because they correspond to the 2.5th and 97.5th percentiles of the results from a smaller number (shown) of permutations than in Analysis 1 (main manuscript, Figure 2), particularly for Analysis 3b. The 95% CI of a normal distribution is indicated by the dotted lines.

Computation of significance threshold (right column). Data are shown as a density plot, indicating the relative proportions (density, *x-axis*) of results throughout the range of maximum p-values (*y-axis*) obtained in a large number (see S3.4) of permutations under the null hypothesis (Test B; dotted line). A plot of the theoretical beta-distribution of these results, whose parameters were estimated using the empirical distribution, is shown as a solid line. The top result for Test A, as well as the 95th percentile of the beta-distribution, corresponding with the $-\log_{10}$(p-value) threshold required to achieve a Type 1 error rate of α=0.05 are indicated by the arrows (see S3.4 for methods).

Figure 3. cont.

Quantile-Quantile Plot **Computation of Significance Threshold**

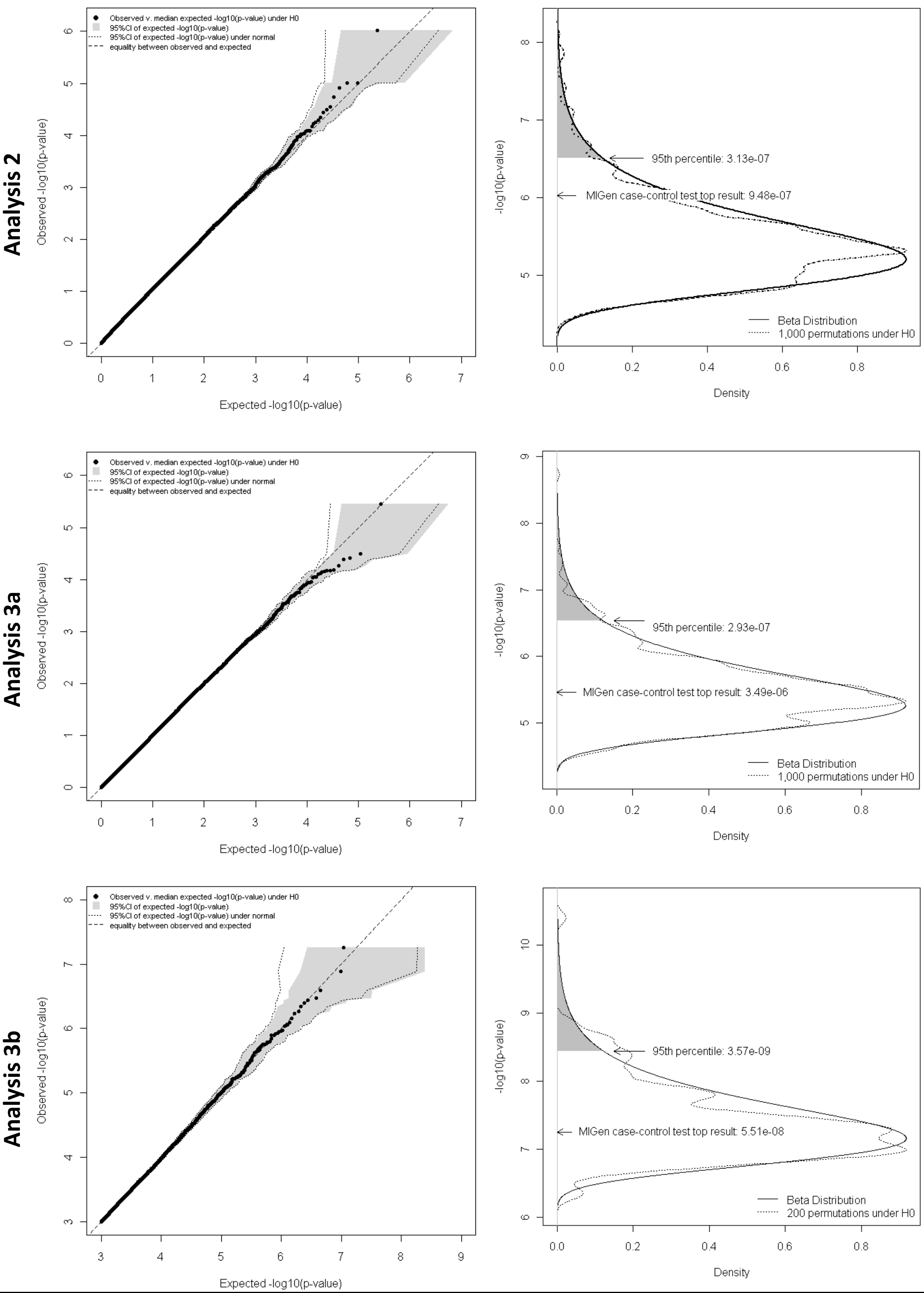

Figure 4. Power computation.

Effect sizes for pairs of SNPs with MAFs between 0.02 and 0.5, under dominant × dominant, recessive × recessive or additive × additive interaction models (Analyses 1, 2, 3a and 3b are shown in rows 1-4, respectively). Allele bins for the SNPs compared are shown on the *x*- and *y-axes*, and the effect size our study has 80% power to detect is shown on the *z-axis*. MAF pairs with missing data (value of 0 on the *z-axis*) indicate instances where the effect size could not be calculated for any of the SNP pairs sampled because of the low frequency of the double rare homozygote. Results are duplicated on either side of a diagonal through the near apex. See S.T3 for raw results.

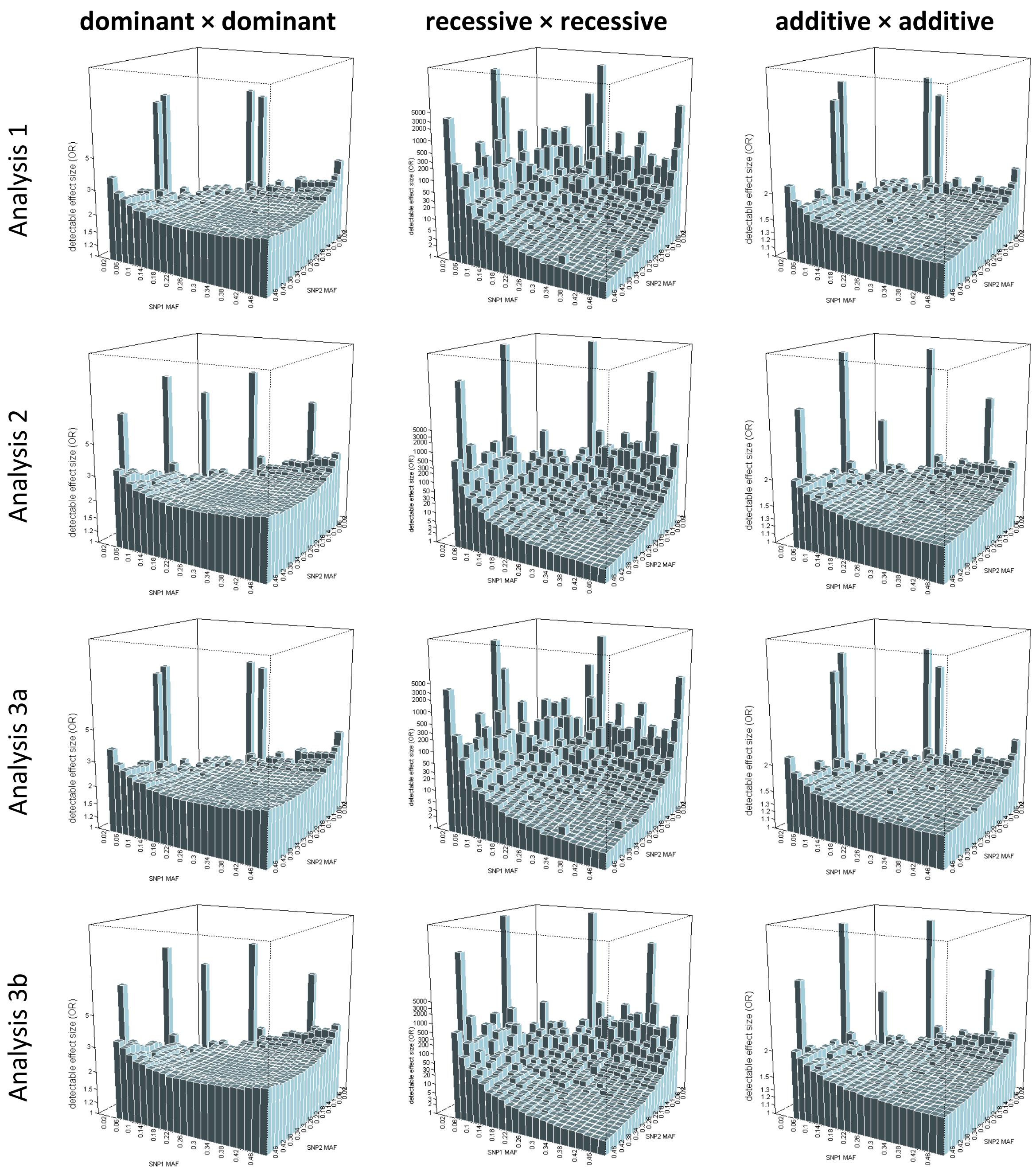

Note 1

Joint case-control/case-only interaction analysis

1. *Introduction*

If we observe a correlation between the alleles or genotypes at two loci in a sample of disease cases, but not in the general population, this would indicate that these variants interact to modulate disease risk. In a case-only interaction analysis, we compute correlation statistics for SNP pairs that are uncorrelated (i.e. in LD) in the general population. Additional power in a case-only analysis is gained from the assumption that the correlation between loci in controls is 0; therefore, this proportion is not an estimation and contains no error.

This approach has the disadvantage that interaction testing cannot be performed between variants that are correlated in the general population. However, this design can be extended to a joint case-control/case-only design, which formally tests for differences in the level of two-locus correlation among cases compared to that among controls, allowing us to also consider SNPs that are correlated in the general population, but that may have a different level of correlation among cases.

2. *Methods*

We have implemented this test by fitting a multinomial regression model, which tests for a significant interaction between case-control status and the genotype of one SNP (SNP 1) as a predictor of the genotype of another SNP (SNP 2); essentially, this compares the level of correlation between the two SNPs among cases to that among controls (4 df).

SNP 2	~	SNP 1 + MI	+	SNP 1 x MI	+	AGE + SEX + PC1 + PC2
3-level response variable		Main effects		Interaction term		Covariates

Similarly to the main case-control analysis reported in this manuscript, we tested for interaction by using a likelihood ratio test to compare the fit of a multinomial regression model containing the SNP 1 x MI status term to an equivalent model lacking this term, with adjustment for age, sex and the first two genetic principal components (PC).

3. *Results*

We compared the results obtained using the case-control test reported in the main manuscript and those obtained using the joint case-control/case-only models, and observed a strong correlation (Pearson correlation coefficient, r^2=0.985 and r^2=0.972 for Analyses 1 and 2 respectively; Figure).

4. *Comments*

The gain in power expected by using the case-only interaction design is likely to be neutralized by the additional error involved in estimating the two-locus correlation among controls in the more general joint case-control/case-only design. Mathematically, the case-control (Test A) and joint case-control/case-only are very similar, with the result that we observe a strong correlation between the results under each design.

The joint case-control/case-only design was previously reported by Zhao *et al.*[60] to be more powerful than a standard logistic regression, but as highlighted by Cordell[61], this might be because of the smaller numbers of degrees of freedom that results from using an allelic test. This allelic test only considers additive interaction models, which may be a disadvantage depending on whether additive × additive models are truly the most common type of gene-gene interaction. Our implementation of the joint case-control/case-only design allows us to capture all interaction models, and also has the advantage of allowing for covariate adjustment.

Note 1, Figure. Comparison of results using the case-control (Test A) and joint case-control/case-only designs.

Analysis 1.

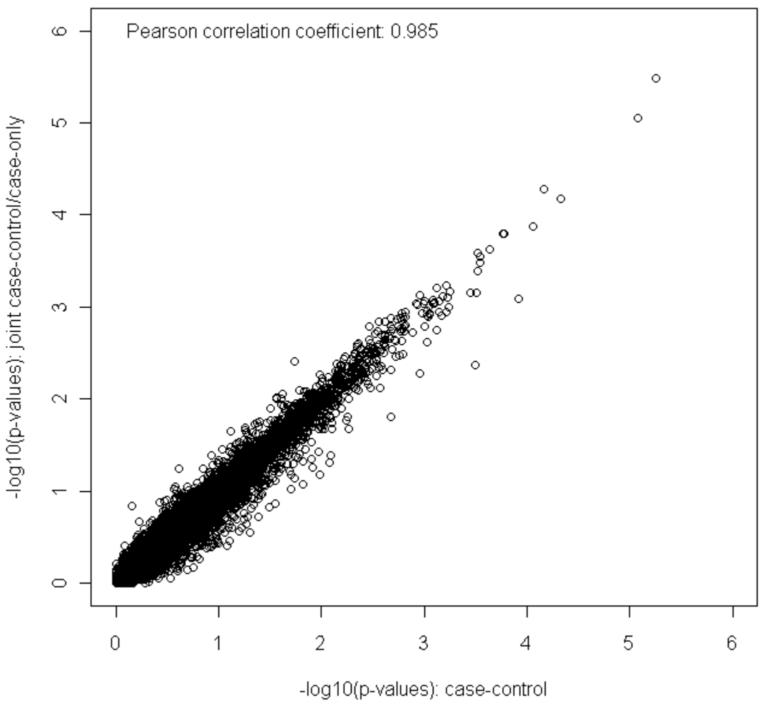

Analysis 2

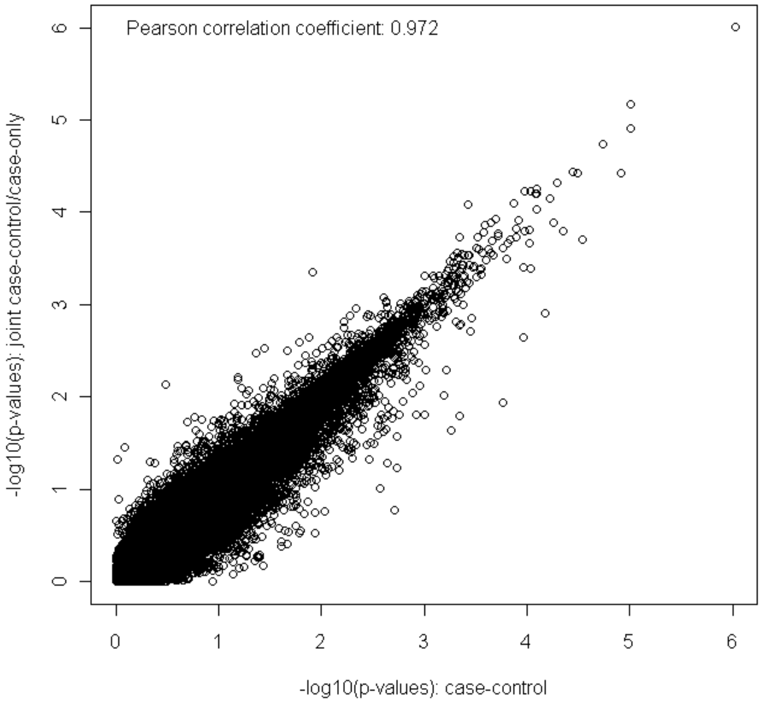

Note 2

Logic Regression analysis

1. *Introduction*

Logic regression was used to perform a preliminary scan for complex interactions, and to investigate whether higher orders of interaction (e.g. pair-wise, 3-way, 4-way, etc.) are more informative in terms of improving the fit of a regression model. Logic regression is an adaptive regression methodology developed mainly for exploring high-order interactions in genomic data[62,63]. It is also useful for predicting the outcome in regression problems based on Boolean combinations of logic variables (for instance a SNP coded as the rare homozygote genotype or not) using logical expressions (e.g. 'AND', 'OR', etc.) (see ref[63] for further details). These combinations are known as logic trees, L.

The order of interactions expressed by a logic tree is given by its size, which corresponds to the number of combinations of SNPs it contains, each connected by a logical expression. Moreover, for complex diseases we may want to simultaneously consider the additive effects of more than one logic tree as potential predictors of the outcome of interest. Thus, we can model these variables (as predictors of the likelihood of a dichotomous outcome, for example) as follows:

$$logit\left(P\left(Y=1;X\right)\right)=b_0+bX+\sum_{i=1}^{j}b_iL_i\ ,$$

where each Lj is a separate logic tree, Y is the outcome (Y=0 for controls, Y=1 for cases) and X denotes covariates (e.g. age, sex, eigen vectors, etc.). Note that, since this technique searches for logical combinations of genetic risk factors, the SNPs being analyzed must necessarily be coded under dominant/recessive models, such that risk may be associated with the presence or absence of either allele.

2. *Methods*

In order to assess the relative gain of information that might be available by exploring higher order interactions, we used a cross-validation approach implemented in the *LogicReg::logreg* function to search for robust models containing up to 5 SNPs distributed across up to 5 logic trees. The sample was partitioned into a training set, in which these models were fit, and a test set, in which the robustness of the best fitting models was assessed. The “quality” of the models under consideration is assessed using

a score function, which in our case (logistic regression of predictors on the dichotomous MI response) reflects the model deviance, where the best fitting models are those that have the lowest total deviance. Having estimated the optimal model type/complexity, we then performed an exhaustive search of the dataset to identify the best fitting scenario (combination of SNPs and logic trees).

We performed this model search among the 242 risk factor SNPs analyzed in Analysis 1, and the 643 SNPs with marginal association with MI that were analyzed in Analysis 3a. We were unable to use the logic regression approach to search for interactions between the SNPs included in Analysis 2 because this consisted of two mutually exclusive sets of SNPs (S.F2), whereas *LogicReg::logreg* is currently restricted to searching for interactions within a single set of SNPs. Moreover, due to computational restrictions, we were unable to perform a joint search of all 6,066 SNPs in Analysis 3b of the main analysis, so we limited this search to a random sample of 2,000 of the Analysis 3b SNPs. All of these analyses were adjusted for sex.

3. *Results*

In the following figures, we present the results of the model search (left column) and the search for the best fitting scenario under the optimal model (right column). The results of the model search are presented as a graph of the average deviances (*y-axis*) of all models tested (black squares), where the best fitting model has the lowest deviance. The number of logic trees in the model is shown in the black squares, and the number of variants distributed across these trees indicated on the *x-axis*.

Analysis 1 – SNPs strongly associated with CAD risk or with classical cardiovascular risk factors.

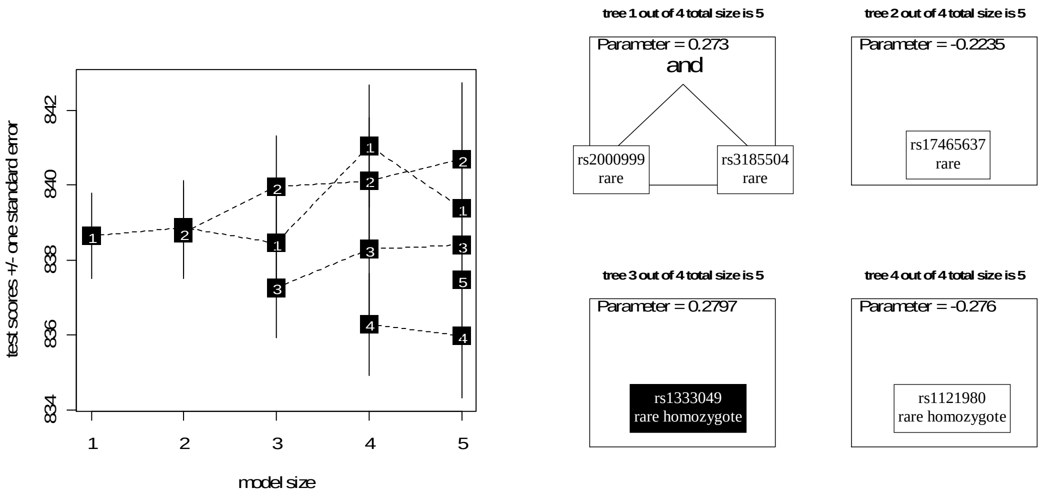

The best scoring model contained 5 variants distributed over 4 logic trees, as follows:

MI risk ~ 0.273[presence of rs2000999_rare AND rs3184504_rare] - 0.223*[presence of rs17465637_rare] + 0.28*[absence of rs1333049_rare homozygote] - 0.276*[presence of rs1121980_rare homozygote]*

These results highlight the fact that model fit is improved primarily by the additive effects of individual variants, and that interaction effects only begin to become relevant when the model is already very complex. Note that, unlike in the subsequent Analyses, addition of multiple single loci significantly improves model fit (i.e. improves the estimation of risk) because these variants are already known to be relevant for cardiovascular risk factors or CAD endpoints.

Analysis 3a – SNPs with modest marginal association with MI in the MIGen study (with p ≤10^{-3}).

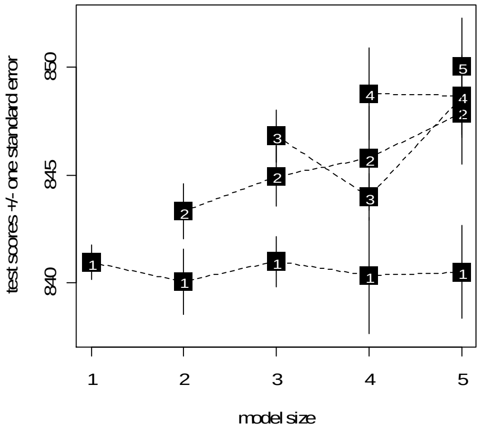

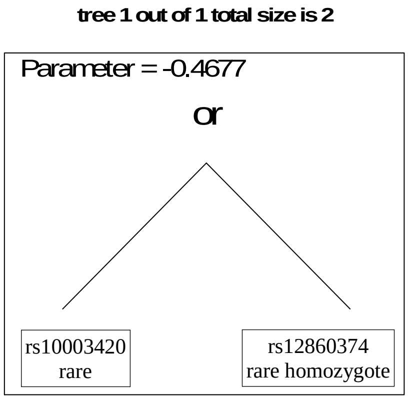

The best scoring model contained 2 variants in a single logic tree, as follows:

MI risk ~ -0.468[presence of rs10003420_rare OR rs12860374_rare homozygote]*

On the basis of these results, we find no evidence to suggest that high-order interactions are important for MI risk. A second order interaction provided the best model fit, but this fit was not significantly better that that of models that consisted of single SNPs or 3-, 4-, or 5-way interaction.

Analysis 3b – A sample of 2000 SNPs with modest marginal association with MI in the MIGen study (with p ≤10^{-2}).

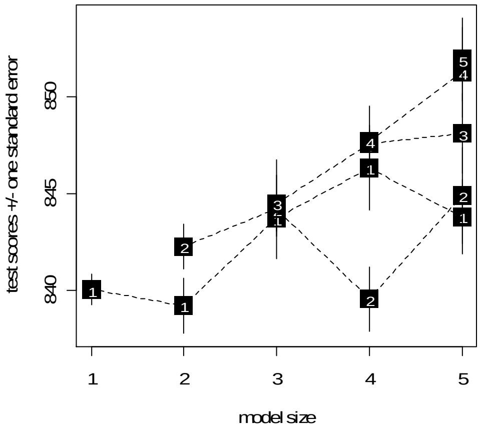

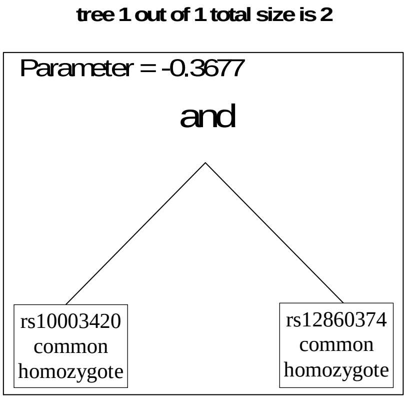

The best scoring model contained 2 variants in a single logic tree, as follows:

MI risk ~ -0.368[presence of rs1887797_common homozygote AND rs31696_common homozygote]*

The results of this analysis are consistent with those from Analysis 3a, in showing no significant evidence to suggest that higher order interactions improve the estimation of disease risk over the information provided by single loci.

4. *Conclusion*

In general, the results of this logic regression analysis are consistent with those of the analysis of gene-gene interactions described in the main manuscript in that they indicate that little additional information is to be gained from these data by exploring pair-wise or higher order interactions.

Appendix 1

MIGen Investigators

MEMBERS

Sekar Kathiresan (leader), Benjamin F Voight, Shaun Purcell, Kiran Musunuru, Diego Ardissino, Pier M Mannucci, Sonia Anand, James C Engert, Nilesh J Samani, Heribert Schunkert, Jeanette Erdmann, Muredach P Reilly, Daniel J Rader, Thomas Morgan, John A Spertus, Monika Stoll, Domenico Girelli, Pascal P McKeown, Chris C Patterson, David S Siscovick, Christopher J O'Donnell, Roberto Elosua, Leena Peltonen, Veikko Salomaa, Stephen M Schwartz, Olle Melander, David Altshuler

Italian Atherosclerosis, Thrombosis and Vascular Biology Study. Diego Ardissino, Pier Angelica Merlini, Carlo Berzuini, Luisa Bernardinelli, Flora Peyvandi, Marco Tubaro, Patrizia Celli, Maurizio Ferrario, Raffaela Fetiveau, Nicola Marziliano, Giorgio Casari, Michele Galli, Flavio Ribichini, Marco Rossi, Francesco Bernardi, Pietro Zonzin, Alberto Piazza, Pier M Mannucci

Heart Attack Risk in Puget Sound. Stephen M Schwartz, David S Siscovick, Jean Yee, Yechiel Friedlander

Registre Gironi del COR. Roberto Elosua, Jaume Marrugat, Gavin Lucas, Isaac Subirana, Joan Sala, Rafael Ramos

Massachusetts General Hospital Premature Coronary Artery Disease Study. Sekar Kathiresan, James B Meigs, Gordon Williams, David M Nathan, Calum A MacRae, Christopher J O'Donnell

FINRISK. Veikko Salomaa, Aki S Havulinna, Leena Peltonen

Malmo Diet and Cancer Study. Olle Melander, Goran Berglund

FUNDING SOURCES

HARPS. The HARPS study was supported by the grants (R01HL056931, P30ES007033) and a contract (N01HD013107) from US National Institutes of Health.

REGICOR. The REGICOR study was partially funded by the Ministerio de Sanidad y Consumo, Instituto de Salud Carlos III (Red HERACLES RD06/0009), the CIBER Epidemiología y Salud Pública, the FIS and AGAUR Generalitat de Catalunya.

Massachusetts General Hospital. The MIGen study was funded by the US National Institutes of Health (NIH) and National Heart, Lung, and Blood Institute's STAMPEED genomics research program through a grant to D.A. S.K. is supported by a Doris Duke Charitable Foundation Clinical Scientist Development Award, a charitable gift from the Fannie E. Rippel Foundation, the Donovan Family Foundation, a career development award from the NIH and the Department of Medicine and Cardiovascular Research Center at Massachusetts General Hospital. J.B.M. is supported by grant K24 DK080140 from the NIH.

Broad Institute. Genotyping was partially funded by The Broad Institute Center for Genotyping and Analysis, which is supported by grant U54 RR020278 from the National Center for Research Resources.

FINRISK. V.S. was supported by the Sigrid Juselius Foundation. L.P. was supported by the Center of Excellence in Complex Disease Genetics of the Academy of Finland, the Nordic Center of Excellence in Disease Genetics and the Finnish Foundation for Cardiovascular Research.

Appendix 2

WTCCC Investigators

MEMBERS

Primary Investigators

David Bentley, Matthew A. Brown, Lon R. Cardon, Mark Caulfield, David G. Clayton, Alistair Compston, Nick Craddock, Panos Deloukas, Peter Donnelly, Martin Farrall, Stephen C. L. Gough, Alistair S. Hall, Andrew T. Hattersley, Adrian V. S. Hill, Dominic P. Kwiatkowski, Christopher G. Mathew, Mark I. McCarthy, Willem H. Ouwehand, Miles Parkes, Marcus Pembrey, Nazneen Rahman, Nilesh J. Samani, Michael R. Stratton, John A. Todd, Jane Worthington

Management Committee

Paul R. Burton, David G. Clayton, Lon R. Cardon, Nick Craddock, Panos Deloukas, Audrey Duncanson, Dominic P. Kwiatkowski, Mark I. McCarthy, Willem H. Ouwehand, Nilesh J. Samani, John A. Todd, Peter Donnelly, (Chair)

Data and Analysis Committee

Jeffrey C. Barrett, Paul R. Burton, Dan Davison, Peter Donnelly, Doug Easton, David Evans, Hin-Tak Leung, Jonathan L. Marchini, Andrew P. Morris, Chris C. A. Spencer, Martin D. Tobin, Lon R. Cardon, (Co-Chair) & David G. Clayton, (Co-Chair)

UK Blood Services and University of Cambridge Controls

Antony P. Attwood, James P. Boorman, Barbara Cant, Ursula Everson, Judith M. Hussey, Jennifer D. Jolley, Alexandra S. Knight, Kerstin Koch, Elizabeth Meech, Sarah Nutland, Christopher V. Prowse, Helen E. Stevens, Niall C. Taylor, Graham R. Walters, Neil M. Walker, Nicholas A. Watkins, Thilo Winzer, John A. Todd & Willem H. Ouwehand

1958 Birth Cohort Controls

Richard W. Jones, Wendy L. McArdle, Susan M. Ring, David P. Strachan, Marcus Pembrey

Coronary Artery Disease

Stephen G. Ball, Anthony J. Balmforth, Jennifer H. Barrett, D. Timothy Bishop, Mark M. Iles, Azhar Maqbool, Nadira Yuldasheva, Alistair S. Hall (Leeds), Peter S. Braund, Paul R. Burton, Richard J. Dixon, Massimo Mangino, Suzanne Stevens, Martin D. Tobin, John R. Thompson, Nilesh J. Samani

DNA, Genotyping, Data QC and Informatics

Suzannah J. Bumpstead, Amy Chaney, Kate Downes, Mohammed J. R. Ghori, Rhian Gwilliam, Sarah E. Hunt, Michael Inouye, Andrew Keniry, Emma King, Ralph McGinnis, Simon Potter, Rathi Ravindrarajah, Pamela Whittaker, Claire Widden, David Withers, Panos Deloukas, Hin-Tak Leung, Sarah Nutland, Helen E. Stevens, Neil M. Walker, John A. Todd

Statistics

Doug Easton, David G. Clayton, Paul R. Burton, Martin D. Tobin, Jeffrey C. Barrett, David Evans, Andrew P. Morris, Lon R. Cardon, Niall J. Cardin, Dan Davison, Teresa Ferreira, Joanne Pereira-Gale, Ingileif B. Hallgrimsdóttir, Bryan N. Howie, Jonathan L. Marchini, Chris C. A. Spencer, Zhan Su, Yik Ying Teo, Damjan Vukcevic, Peter Donnelly

FUNDING SOURCES

The principal funder of this project was the Wellcome Trust. Case collections were funded by: Arthritis Research Campaign, BDA Research, British Heart Foundation, British Hypertension Society, Diabetes UK, Glaxo-Smith Kline Research and Development, Juvenile Diabetes Research Foundation, National Association for Colitis and Crohn's disease, SHERT (The Scottish Hospitals Endowment Research Trust), St Bartholomew's and The Royal London Charitable Foundation, UK Medical Research Council, UK NHS R&D and the Wellcome Trust. Statistical analyses were funded by a Commonwealth Scholarship, EU, EPSRC, Fundação para a Ciência e a Tecnologia (Portugal), National Institutes of Health, National Science Foundation and the Wellcome Trust. For the 1958 Birth Cohort, venous blood collection was funded by the UK Medical Research Council and cell-line production, DNA extraction and processing by the Juvenile Diabetes Research Foundation and the Wellcome Trust.

References

1. Myocardial Infarction Genetics Consortium (2009) Genome-wide association of early-onset myocardial infarction with single nucleotide polymorphisms and copy number variants. Nat Genet 41: 334-341.
2. Li Y, Willer CJ, Ding J, Scheet P, Abecasis GR (2010) MaCH: using sequence and genotype data to estimate haplotypes and unobserved genotypes. Genet Epidemiol 34: 816-834.
3. Wellcome Trust Case Control Consortium (2007) Genome-wide association study of 14,000 cases of seven common diseases and 3,000 shared controls. Nature 447: 661-678.
4. Hindorff LA, Junkins HA, Mehta JP, Manolio TA (2009) A Catalog of Published Genome-Wide Association Studies.
5. Saxena R, Hivert MF, Langenberg C, Tanaka T, Pankow JS, *et al*. (2010) Genetic variation in GIPR influences the glucose and insulin responses to an oral glucose challenge. Nat Genet 42: 142-148.
6. Dupuis J, Langenberg C, Prokopenko I, Saxena R, Soranzo N, *et al*. (2010) New genetic loci implicated in fasting glucose homeostasis and their impact on type 2 diabetes risk. Nat Genet 42: 105-116.
7. Chasman DI, Pare G, Zee RY, Parker AN, Cook NR, *et al*. (2008) Genetic loci associated with plasma concentration of low-density lipoprotein cholesterol, high-density lipoprotein cholesterol, triglycerides, apolipoprotein A1, and Apolipoprotein B among 6382 white women in genome-wide analysis with replication. Circ Cardiovasc Genet 1: 21-30.
8. R Development Core Team (2010) R: A language and environment for statistical computing. R Foundation for Statistical Computing, Vienna, Austria. URL http://www.R-project.org/-
9. Purcell S, Neale B, Todd-Brown K, Thomas L, Ferreira MA, *et al*. (2007) PLINK: a tool set for whole-genome association and population-based linkage analyses. Am J Hum Genet 81: 559-575.
10. Li W and Reich J (2000) A complete enumeration and classification of two-locus disease models. Hum Hered 50: 334-349.
11. Marchini J, Donnelly P, Cardon LR (2005) Genome-wide strategies for detecting multiple loci that influence complex diseases. Nat Genet 37: 413-417.
12. Kathiresan S (2009) Lp(a) lipoprotein redux--from curious molecule to causal risk factor. N Engl J Med 361: 2573-2574.
13. Clarke R, Peden JF, Hopewell JC, Kyriakou T, Goel A, *et al*. (2009) Genetic variants associated with Lp(a) lipoprotein level and coronary disease. N Engl J Med 361: 2518-2528.
14. Tregouet DA, Konig IR, Erdmann J, Munteanu A, Braund PS, *et al*. (2009) Genome-wide haplotype association study identifies the SLC22A3-LPAL2-LPA gene cluster as a risk locus for coronary artery disease. Nat Genet 41: 283-285.
15. Lake SL, Lyon H, Tantisira K, Silverman EK, Weiss ST, *et al*. (2003) Estimation and tests of haplotype-environment interaction when linkage phase is ambiguous. Hum Hered 55: 56-65.
16. Kathiresan S, Willer CJ, Peloso GM, Demissie S, Musunuru K, *et al*. (2009) Common variants at 30 loci contribute to polygenic dyslipidemia. Nat Genet 41: 56-65.
17. Teslovich TM, Musunuru K, Smith AV, Edmondson AC, Stylianou IM, *et al*. (2010) Biological, clinical and population relevance of 95 loci for blood lipids. Nature 466: 707-713.
18. Ober C, Nord AS, Thompson EE, Pan L, Tan Z, *et al*. (2009) Genome-wide association study of plasma lipoprotein(a) levels identifies multiple genes on chromosome 6q. J Lipid Res 50: 798-806.
19. Samani NJ, Erdmann J, Hall AS, Hengstenberg C, Mangino M, *et al*. (2007) Genomewide association analysis of coronary artery disease. N Engl J Med 357: 443-453.
20. Loos RJ, Lindgren CM, Li S, Wheeler E, Zhao JH, *et al*. (2008) Common variants near MC4R are associated with fat mass, weight and risk of obesity. Nat Genet 40: 768-775.
21. Kathiresan S, Melander O, Guiducci C, Surti A, Burtt NP, *et al*. (2008) Six new loci associated with blood low-density lipoprotein cholesterol, high-density lipoprotein cholesterol or triglycerides in humans. Nat Genet 40: 189-197.
22. Schunkert H, Konig IR, Kathiresan S, Reilly MP, Assimes TL, *et al*. (2011) Large-scale association analysis identifies 13 new susceptibility loci for coronary artery disease. Nat Genet 43: 333-338.
23. Ehret GB, Munroe PB, Rice KM, Bochud M, Johnson AD, *et al*. (2011) Genetic variants in novel pathways influence blood pressure and cardiovascular disease risk. Nature 478: 103-109.
24. Levy D, Ehret GB, Rice K, Verwoert GC, Launer LJ, *et al*. (2009) Genome-wide association study of blood pressure and hypertension. Nat Genet
25. Kamatani Y, Matsuda K, Okada Y, Kubo M, Hosono N, *et al*. (2010) Genome-wide association study of hematological and biochemical traits in a Japanese population. Nat Genet 42: 210-215.
26. Zeggini E, Scott LJ, Saxena R, Voight BF, Marchini JL, *et al*. (2008) Meta-analysis of genome-wide association data and large-scale replication identifies additional susceptibility loci for type 2 diabetes. Nat Genet 40: 638-645.
27. Willer CJ, Sanna S, Jackson AU, Scuteri A, Bonnycastle LL, *et al*. (2008) Newly identified loci that influence lipid concentrations and risk of coronary artery disease. Nat Genet 40: 161-169.

28. Newton-Cheh C, Johnson T, Gateva V, Tobin MD, Bochud M, *et al*. (2009) Genome-wide association study identifies eight loci associated with blood pressure. Nat Genet 41: 666-676.
29. Thorleifsson G, Walters GB, Gudbjartsson DF, Steinthorsdottir V, Sulem P, *et al*. (2009) Genome-wide association yields new sequence variants at seven loci that associate with measures of obesity. Nat Genet 41: 18-24.
30. Qi L, Cornelis MC, Kraft P, Stanya KJ, Linda Kao WH, *et al*. (2010) Genetic variants at 2q24 are associated with susceptibility to type 2 diabetes. Hum Mol Genet 19: 2706-2715.
31. Aulchenko YS, Ripatti S, Lindqvist I, Boomsma D, Heid IM, *et al*. (2009) Loci influencing lipid levels and coronary heart disease risk in 16 European population cohorts. Nat Genet 41: 47-55.
32. Sabatti C, Service SK, Hartikainen AL, Pouta A, Ripatti S, *et al*. (2009) Genome-wide association analysis of metabolic traits in a birth cohort from a founder population. Nat Genet 41: 35-46.
33. Tsai FJ, Yang CF, Chen CC, Chuang LM, Lu CH, *et al*. (2010) A genome-wide association study identifies susceptibility variants for type 2 diabetes in Han Chinese. PLoS Genet 6: e1000847-
34. Chambers JC, Elliott P, Zabaneh D, Zhang W, Li Y, *et al*. (2008) Common genetic variation near MC4R is associated with waist circumference and insulin resistance. Nat Genet 40: 716-718.
35. Voight BF, Scott LJ, Steinthorsdottir V, Morris AP, Dina C, *et al*. (2010) Twelve type 2 diabetes susceptibility loci identified through large-scale association analysis. Nat Genet 42: 579-589.
36. Sandhu MS, Waterworth DM, Debenham SL, Wheeler E, Papadakis K, *et al*. (2008) LDL-cholesterol concentrations: a genome-wide association study. Lancet 371: 483-491.
37. Thorgeirsson TE, Gudbjartsson DF, Surakka I, Vink JM, Amin N, *et al*. (2010) Sequence variants at CHRNB3-CHRNA6 and CYP2A6 affect smoking behavior. Nat Genet 42: 448-453.
38. Erdmann J, Grosshennig A, Braund PS, Konig IR, Hengstenberg C, *et al*. (2009) New susceptibility locus for coronary artery disease on chromosome 3q22.3. Nat Genet 41: 280-282.
39. Willer CJ, Speliotes EK, Loos RJ, Li S, Lindgren CM, *et al*. (2009) Six new loci associated with body mass index highlight a neuronal influence on body weight regulation. Nat Genet 41: 25-34.
40. Tobacco and Genetics Consortium (2010) Genome-wide meta-analyses identify multiple loci associated with smoking behavior. Nat Genet 42: 441-447.
41. Zeggini E, Weedon MN, Lindgren CM, Frayling TM, Elliott KS, *et al*. (2007) Replication of genome-wide association signals in UK samples reveals risk loci for type 2 diabetes. Science 316: 1336-1341.
42. Meyre D, Delplanque J, Chevre JC, Lecoeur C, Lobbens S, *et al*. (2009) Genome-wide association study for early-onset and morbid adult obesity identifies three new risk loci in European populations. Nat Genet 41: 157-159.
43. Tarasov KV, Sanna S, Scuteri A, Strait JB, Orru M, *et al*. (2009) COL4A1 is associated with arterial stiffness by genome-wide association scan. Circ Cardiovasc Genet 2: 151-158.
44. Lowe JK, Maller JB, Pe'er I, Neale BM, Salit J, *et al*. (2009) Genome-wide association studies in an isolated founder population from the Pacific Island of Kosrae. PLoS Genet 5: e1000365-
45. Lindgren CM, Heid IM, Randall JC, Lamina C, Steinthorsdottir V, *et al*. (2009) Genome-wide association scan meta-analysis identifies three Loci influencing adiposity and fat distribution. PLoS Genet 5: e1000508-
46. Kooner JS, Chambers JC, Aguilar-Salinas CA, Hinds DA, Hyde CL, *et al*. (2008) Genome-wide scan identifies variation in MLXIPL associated with plasma triglycerides. Nat Genet 40: 149-151.
47. Rung J, Cauchi S, Albrechtsen A, Shen L, Rocheleau G, *et al*. (2009) Genetic variant near IRS1 is associated with type 2 diabetes, insulin resistance and hyperinsulinemia. Nat Genet 41: 1110-1115.
48. Pollin TI, Damcott CM, Shen H, Ott SH, Shelton J, *et al*. (2008) A null mutation in human APOC3 confers a favorable plasma lipid profile and apparent cardioprotection. Science 322: 1702-1705.
49. Heard-Costa NL, Zillikens MC, Monda KL, Johansson A, Harris TB, *et al*. (2009) NRXN3 is a novel locus for waist circumference: a genome-wide association study from the CHARGE Consortium. PLoS Genet 5: e1000539-
50. Prokopenko I, Langenberg C, Florez JC, Saxena R, Soranzo N, *et al*. (2009) Variants in MTNR1B influence fasting glucose levels. Nat Genet 41: 77-81.
51. Takeuchi F, Serizawa M, Yamamoto K, Fujisawa T, Nakashima E, *et al*. (2009) Confirmation of multiple risk Loci and genetic impacts by a genome-wide association study of type 2 diabetes in the Japanese population. Diabetes 58: 1690-1699.
52. Saxena R, Voight BF, Lyssenko V, Burtt NP, de Bakker PI, *et al*. (2007) Genome-wide association analysis identifies loci for type 2 diabetes and triglyceride levels. Science 316: 1331-1336.
53. Scott LJ, Mohlke KL, Bonnycastle LL, Willer CJ, Li Y, *et al*. (2007) A genome-wide association study of type 2 diabetes in Finns detects multiple susceptibility variants. Science 316: 1341-1345.
54. Chambers JC, Zhang W, Zabaneh D, Sehmi J, Jain P, *et al*. (2009) Common genetic variation near melatonin receptor MTNR1B contributes to raised plasma glucose and increased risk of type 2 diabetes among Indian Asians and European Caucasians. Diabetes 58: 2703-2708.

55. Heid IM, Boes E, Muller M, Kollerits B, Lamina C, *et al*. (2008) Genome-wide association analysis of high-density lipoprotein cholesterol in the population-based KORA study sheds new light on intergenic regions. Circ Cardiovasc Genet 1: 10-20.
56. Bouatia-Naji N, Rocheleau G, Van Lommel L, Lemaire K, Schuit F, *et al*. (2008) A polymorphism within the G6PC2 gene is associated with fasting plasma glucose levels. Science 320: 1085-1088.
57. Liu XG, Tan LJ, Lei SF, Liu YJ, Shen H, *et al*. (2009) Genome-wide association and replication studies identified TRHR as an important gene for lean body mass. Am J Hum Genet 84: 418-423.
58. Yasuda K, Miyake K, Horikawa Y, Hara K, Osawa H, *et al*. (2008) Variants in KCNQ1 are associated with susceptibility to type 2 diabetes mellitus. Nat Genet 40: 1092-1097.
59. Unoki H, Takahashi A, Kawaguchi T, Hara K, Horikoshi M, *et al*. (2008) SNPs in KCNQ1 are associated with susceptibility to type 2 diabetes in East Asian and European populations. Nat Genet 40: 1098-1102.
60. Zhao J, Jin L, Xiong M (2006) Test for interaction between two unlinked loci. Am J Hum Genet 79: 831-845.
61. Cordell HJ (2009) Detecting gene-gene interactions that underlie human diseases. Nat Rev Genet 10: 392-404.
62. Kooperberg C and Ruczinski I (2005) Identifying interacting SNPs using Monte Carlo logic regression. Genet Epidemiol 28: 157-170.
63. Ruczinski I, Kooperberg C, LeBlanc M (2003) Logic regression. J Comput Graph Stat 12: 475-511.
